# Development and evaluation of a web-based diet quality screener for vegans (VEGANScreener): a cross-sectional, observational, multicenter, clinical study

**DOI:** 10.3389/fnut.2024.1438740

**Published:** 2024-10-22

**Authors:** Tooba Asif, Anna Ouřadová, Ainara Martínez Tabar, Vanessa Bullón-Vela, Sandra Müller, Joelina Dietrich, Vanessa Keller, Marina Heniková, Eliška Selinger, Isabelle Herter-Aeberli, Maria Wakolbinger, Willem De Keyzer, Wendy Van Lippevelde, Monika Cahová, Leonie H. Bogl, Marek Kuzma, Maira Bes-Rastrollo, Stefaan De Henauw, Markus Keller, Selma Kronsteiner-Gicevic, Eva Schernhammer, Jan Gojda

**Affiliations:** ^1^Faculty of Medicine and Health Sciences, Department of Public Health and Primary Care, Ghent University, Ghent, Belgium; ^2^Department of Internal Medicine, Third Faculty of Medicine, Charles University, and Kralovske Vinohrady University Hospital and Third Faculty of Medicine, Charles University, Prague, Czechia; ^3^Department of Preventive Medicine and Public Health, University of Navarra-IdiSNA, Pamplona, Spain; ^4^Research Institute for Plant-Based Nutrition, Biebertal, Germany; ^5^Laboratory of Nutrition and Metabolic Epigenetics, Institute of Food, Nutrition and Health, ETH Zürich, Zürich, Switzerland; ^6^Department of Social and Preventive Medicine, Center for Public Health, Medical University of Vienna, Vienna, Austria; ^7^HOGENT University of Applied Sciences and Arts, Ghent, Belgium; ^8^Faculty of Economics and Business, Department of Public Health and Primary Care, Ghent University, Ghent, Belgium; ^9^Institute for Clinical and Experimental Medicine, Prague, Czechia; ^10^Department Nutrition and Dietetics, Faculty of Health Professions, Bern University of Applied Sciences, Bern, Switzerland; ^11^Institute for Molecular Medicine Finland (FIMM), University of Helsinki, Helsinki, Finland; ^12^Institute of Microbiology of the CAS, Prague, Czechia; ^13^CIBERobn, Instituto de Salud Carlos III, Madrid, Spain; ^14^Department of Epidemiology, Center for Public Health, Medical University of Vienna, Vienna, Austria; ^15^Department of Nutritional Sciences, Faculty of Life Sciences, University of Vienna, Vienna, Austria; ^16^Department of Epidemiology, Harvard T.H. Chan School of Public Health, Boston, MA, United States

**Keywords:** nutrition, plant-based diet, vegan diet, diet quality, diet assessment, diet screener

## Abstract

Consumption of plant-based diets, including vegan diets, necessitates attention to the quality of the diet for the prevention and early detection of nutritional deficiencies. Within the VEGANScreener project, a unique brief screening tool for the assessment and monitoring of diet quality among vegans in Europe was developed. To provide a standardized tool for public use, a clinical study will be conducted to evaluate the VEGANScreener against a reference dietary assessment method and nutritional biomarkers. An observational study is set to include 600 participants across five European sites – Belgium, Czech Republic, Germany, Spain, and Switzerland. In total, 400 self-reported vegans (≥2 years on a vegan diet), and 170 self-reported omnivore controls will be examined, aged between 18 and 65 years, with males and females being equally represented in a 1:1 ratio for two age groups (18–35 and 36–65 years). Participants with diseases affecting metabolism and intestinal integrity will be excluded. The clinical assessment will include a structured medical history, along with taking blood pressure and anthropometric measurements. Blood and urine will be sampled and analyzed for a set of dietary biomarkers. Metabolomic analyses will be conducted to explore potential novel biomarkers of vegan diet. Moreover, saliva samples will be collected to assess the metabolome and the microbiome. Participants will receive instructions to complete a nonconsecutive 4-day diet record, along with the VEGANScreener, a socio-demographic survey, a well-being survey, and a FFQ. To evaluate reproducibility, the VEGANScreener will be administered twice over a three-weeks period. Among vegans, the construct validity and criterion validity of the VEGANScreener will be analyzed through associations of the score with nutrient and food group intakes, diet quality scores assessed from the 4-day diet records, and associations with the dietary biomarkers. Secondary outcomes will include analysis of dietary data, metabolomics, and microbiomes in all participants. Major nutrient sources and variations will be assessed in the sample. Exploratory metabolomic analysis will be performed using multivariable statistics and regression analysis to identify novel biomarkers. Standard statistical models will be implemented for cross-sectional comparisons of geographical groups and vegans versus omnivores.

## 1 Introduction

Diets that minimize the consumption of animal-based products, including vegan diets, are rising in developed countries worldwide, with European countries following this trend (1, 2). Veganism encompasses the complete avoidance of food and lifestyle items derived from animals. Initially, it was closely associated with concerns for animal welfare and ethical considerations regarding animal rights (3). Nowadays, an increasing number of individuals are adopting vegan diets, largely motivated by environmental consciousness and the perception that plant-based diets are a more sustainable choice. Additionally, health considerations are also significant drivers behind the adoption of veganism (4–6).

Vegan diets are linked to increased consumption of numerous nutrients primarily found in plant-based foods, including dietary fiber, phytochemicals, various vitamins, minerals (such as vitamins C, thiamin, pyridoxine, vitamin E, folate, and magnesium), as well as polyunsaturated fatty acids like alpha-linolenic acid (ALA) (7, 8). Additionally, they tend to exhibit a lower glycemic load compared to omnivorous diets (8, 9). Embracing a healthy vegan diet is associated with several favorable health outcomes, marked by enhancements in cardiometabolic health, including reduced levels of low-density lipoprotein (LDL) cholesterol and apolipoprotein B (apo B), improved glycemic control characterized by lower fasting glucose and glycated hemoglobin (HbA1c), and substantial weight loss (10–13). The potential of the vegan diet to facilitate weight loss and effective weight management is of particular interest, especially considering the escalating global prevalence of obesity and metabolic syndrome (14).

Vegan diets as the most restrictive type of plant-based diets are especially prone to nutritional deficiencies in some key nutrients, such as the vitamins; riboflavin, niacin, B12 and D, and the minerals; iodine, zinc, calcium, iron and selenium, as well as the polyunsaturated fatty acids; docosahexaenoic acid (DHA) and eicosapentaenoic acid (EPA), and the amino acid; lysine (7, 8, 15–17). These essential nutrients are primarily derived from animal-based sources or exhibit higher bioavailability in such sources. Notably, one of the most extensively debated concerns associated with vegan diets is their impact on bone health. Vegans may face an elevated risk of accelerated bone turnover, decreased bone mass, and an increased susceptibility to fractures (18). These effects are often attributed to lower levels of calcium and vitamin D. It is important to emphasize, however, that these findings are largely based on evidence with low or very low levels of certainty (10).

The rise of veganism has also led to numerous start-ups and innovations in various industries. These innovations aim to provide sustainable and ethical alternatives to traditional animal-derived products. In particular, the food industry has seen a significant increase in vegan offerings, which include plant-based meat, dairy, and egg alternatives. However, the inclusion of novel foods in a vegan diet raises several potential concerns that merit thorough scientific investigation. These concerns include allergenicity, potential nutrient deficiencies, digestive issues, food safety considerations, consumer acceptability, and overall palatability (19).

The growing popularity of vegan diets is accompanied by increasing diversity. The quality of such diets can vary in terms of individual’s intake of critical nutrients, novel foods, processed meat alternatives, and dairy substitutes. Several types of specific dietary patterns among vegans have been identified (20, 21). Of note, the consumption of ultra-processed foods (UPFs) and the energy intake from UPFs was higher among vegans than omnivores (22). These findings highlight the need to distinguish between “healthy” plant-based diets, which focus on whole and raw foods and “unhealthy” plant-based diets characterized by high intake of ultra-processed or energy-dense and nutrient poor foods and beverages high in added sugar (22–24). In this context, evidence-based screening tools to help evaluate diet quality and guide dietary choices for both the vegan population and healthcare professionals are urgently needed.

The VEGANScreener project is an ERA-NET-funded collaboration of five scientific partners from European countries (Austria, Belgium, Czech Republic, Spain, and Germany), with additional partners from the US and Switzerland. Its principal objective is to develop and evaluate a brief quick-to-use diet quality screening tool for the European vegan population (VEGANScreener). The following clinical study is part of the evaluation process. To this end, the primary objective of the study is to assess the construct validity and criterion validity for associations of the VEGANScreener score with nutrient and food group intakes from reference dietary intake methods, i.e., diet quality scores assessed from the 4-day diet records, and associations with the concentration and recovery biomarkers. Furthermore, the study encompasses secondary objectives. These include: examining the associations between dietary biomarkers with biomarkers of disease, as well as anthropometric measures in all participants; comparison of these results with the geographical origin of the groups; detailed dietary data analysis (i.e., to identify main nutrient sources, variations in diet quality, dietary patterns and dietary biomarkers between groups); metabolomics analysis (i.e., blood and saliva) to identify novel concentration biomarkers of dietary intake and nutritional adequacy, and the microbiome analysis from saliva to identify diet-specific omics signatures.

## 2 Methods and analysis

### 2.1 Design

**Figure 1:**
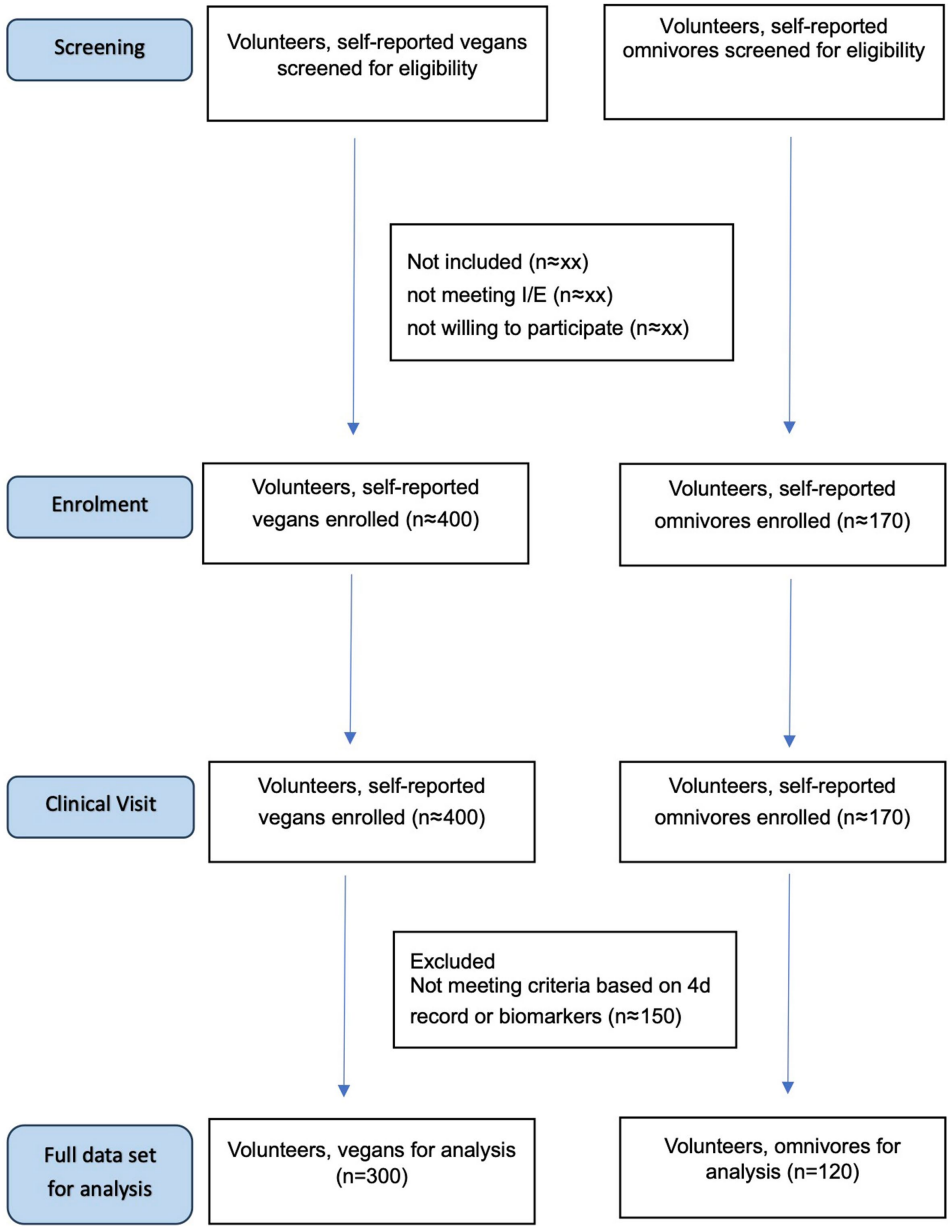
STROBE flow diagram.

The VEGANScreener clinical study is an observational, cross-sectional, multicenter study. The study follows the standard protocols of STROBE guidelines, and the diagram of participant flow is depicted in Figure 1.

### 2.2 Study setting and timeline

Data collection will be conducted in five geographically distinct European areas at the following academic institutions: Research Institute for Plant-based Nutrition, Biebertal/Gießen, Germany; Královské Vinohrady University Hospital, Prague, Czech Republic; IdiSNA-University of Navarra, Pamplona, Spain; Department of Public Health and Primary Care, Ghent University, Belgium; ETH Zurich, Laboratory for Nutrition and Metabolic Epigenetics, Institute of Food, Nutrition and Health, Zurich, Switzerland. Participant enrolment takes place between April 1, 2023 and January 31, 2024.

### 2.3 Eligibility criteria and recruitment

Vegans and a smaller group of omnivores will be recruited for the study. The inclusion criteria are (1) self-reported vegans (≥2 years on a vegan diet; vegan diet defined as not consuming any dietary animal products more often than once/month, honey excluded) or (2) self-reported omnivores (≥2 years on an omnivorous diet; consuming on average daily at least 5 times/week meat or meat products). Both males and females (1:1 ratio) will be included aged 18 to 65 years (1:1 ratio 18–35, and 36–65). The exclusion criteria are as follows: (1) history of a disease known to affect intermediary metabolism (e.g., any diabetes on treatment, i.e., medication or lifestyle recommendations, liver disease, chronic kidney disease, thyropathies, cancer); (2) BMI ≥ 30 kg/m2; (3) history of disease of intestinal integrity (i.e., inflammatory bowel disease, chronic pancreatitis, other malabsorption, etc.) and (4) currently pregnant or breastfeeding. The sample of omnivores will be recruited in several study sites to serve as a comparison group for secondary outcomes of the study (i.e., metabolomics and microbiome analysis).

Social media, newspaper advertisements, vegan societies, direct contact with vegan persons in outpatients dept. and direct invitational emails to previous research participants will be used to reach out to the target population. Potential volunteers will go through an online eligibility check (See SOP 1 in Supplementary materials). Track of records for screening, screening failures and enrolment rates will be recorded in REDCap, a secure web application for building and managing online surveys and databases.

### 2.4 Clinical examination

The clinical visit will be performed at each site. A detailed clinical examination consisting of structured medical history (see SOP 2 in Supplementary materials), measurements for blood pressure, heart rate, weight, height, hip, and waist circumference will be carried out by trained professionals. All clinical parameters will be measured three times, and the average value will be used for the final calculations (see SOP 3 in Supplementary materials). Data will be stored in REDCap.

### 2.5 Laboratory parameters

After an overnight fast (12 h) venous blood sampling from the antecubital vein will be performed for the laboratory evaluations: Tubes will be collected, centrifuged and aliquots will be coded, kept refrigerated, and stored at −80°C during the same morning until being analyzed (see SOP 4 in Supplementary materials). Similarly, saliva samples will be collected at the study center. Study participants are asked to not eat, drink, brush, and/or floss their teeth or smoke 12 h before the sampling (see SOP 5 in Supplementary materials). Spot urine samples will be collected at the initial clinical visit. 24-h urine samples will be collected at home using the provided containers. Collected urine will be delivered at visit 2 (see SOP 6 in Supplementary materials). Biomarkers of interest are depicted in Table 1. The majority of these are concentration biomarkers reflecting nutritional status. As a recovery biomarker to validate protein intake a 24-h nitrogen output will be assessed. All the parameters will be analyzed in ISO-certified institutional laboratories.

**Table 1:** Biomarkers of interest.

Category	Tests
Blood	Minerals: Zinc, selenium, magnesium, calcium
	Iron metabolism: Hemoglobin, ferritin, soluble transferrin receptors
	Vitamins: Folate, vitamin B2, vitamin C, vitamin D, holo-transcobalamin, MMA, homocysteine
	Lipids: Cholesterol, HDL, LDL, TAG; fatty acids, serum EPA and DHA
	Glucose, hsCRP, uric acid, creatinine
24-h urine	Iodine, urea, sodium, potassium
Spot urine	Iodine, calcium, creatinine

MMA, Methylmalonic acid; HDL, High-density lipoproteins; LDL, Low-density lipoproteins; TAG, Triacylglycerols; EPA, Eicosapentoenoic acid; DHA, Docosahexaenoic acid; hsCRP, high sensitivity C-reactive protein.

### 2.6 Dietary intake assessment

Each participant will complete a 4-day prospective diet record (see SOP 7 in Supplementary materials) comprising four non-consecutive days distributed over the next 2 weeks after the study center visit (three weekdays and one weekend day). The days will be agreed upon between the clinician and participant during the clinical visit. Participants will receive verbal instructions on how to keep diet records; they will also receive written instructions, paper diet record forms, and a digital scale; finally, they will be provided support via telephone/email throughout the data collection process. Participants will be given the option to record their responses using a paper form or an electronic form provided via MyCap (this form will not be superior to the paper form; it will simply serve as a digital notepad). All consumed foods, beverages, and supplements will be recorded, including preparation methods, brand names, and exact amounts consumed. Participants will be instructed to take photos of their meals and food packaging. They will also be asked to report any health issues during the days of record-keeping and whether their reported intake reflected their usual diet. Nutrient and food intakes will be estimated *post hoc* from raw food data (i.e., diet records) using diet assessment software and a combination of food composition databases (FCDBs) including the German Food Code and Nutrient Database (BLS), the Danish Food Composition Databank (Frida), the Czech Food Composition Database (NutriCZ), the USDA National Nutrient Database for Standard Reference, the USDA Branded Food Products Database, the Swiss Food Composition Database, and the Spanish Food Composition Database. We will intend to use a hierarchical stepwise approach to data sourcing. German BLS database (BLS, Bundeslebensmittelschlüssel, Max Rubner-Institut Federal Research Institute of Nutrition and Food, Germany) includes a large number of food codes and a wide number of nutrients and will therefore be used as the initial data source for all generic food items (e.g., fruits and vegetables, nuts, legumes, or grains). The BLS, however, might not include data on some novel vegan products or some local food items specific to participating countries (e.g., data for chicory in the BLS might be for Belgian endive and not for *Cichorium endivia* var. crispum commonly consumed in Spain), therefore at a second step our food database will be supplemented with data from the national databases (e.g., Spanish, Czech). Data on vegan products currently not in the BLS and not country-specific will be sourced from the Danish food composition database (Frida, National Food Institute, Technical University of Denmark, Denmark), the USDA Branded Food Products Database, and participant-provided back-of-packaging photographs (i.e., ‘branded goods’). Novel vegan products, not available in the mentioned databases, will be created as recipes based on the ingredients and nutrients on the back-of-packaging information. Finally, composite foods/recipes will be created by trained dietitians using food codes from the databases above. Data on the use of supplements will be separately analysed and added to nutrients estimated from foods during analysis, when appropriate.

### 2.7 VEGANScreener

The VEGANScreener, a brief diet quality screening tool for vegans (see SOP 8 in Supplementary materials), consists of 29 questions and one sub-question; of these, 17 questions focus on the intake of food groups and nutrients whose intake should be encouraged (e.g., wholegrain bread, bun, roll, crisp or crackers) and 12 (+ one sub-question) on intake of food groups that should be limited (e.g., white bread, bun or roll); 24 food-based (e.g., sugar-sweetened beverages) and five nutrient/supplement-based (e.g., vitamin B12 supplement).

Diet quality is a multidimensional construct developed in nutritional epidemiology to assess dietary patterns and their associations with health outcomes or the effectiveness of dietary interventions (25, 26). In line with this concept, the VEGANScreener tool will assess vegan diets in 4 dimensions: adequacy, balance, moderation and variety of healthy dietary components (27). The development process of the VEGANScreener diet quality assessment tool is beyond the focus of this protocol and is further described in detail in a separate article (28).

### 2.8 Online surveys

Several online surveys will be completed in REDCap by the participants.

Each participant will be completing a general survey (see SOP 9 in Supplementary materials) focusing on basic sociodemographic data, and dietary and lifestyle habits. As part of the general survey, each subject will self-administer the International Physical Activity Questionnaire (IPAQ) in its short form. This measure assesses the types of intensity of physical activity and sitting time in a population aged 15–69 years as part of their daily lives. It estimates the total physical activity in metabolic equivalent (MET)-min/week and time spent sitting (29).

After completing the VEGANScreener, the general questionnaire, and the 4-day diet record each participant will optionally complete the following assessments:

A physiological well-being and mental health assessment questionnaire (see SOP 10 in Supplementary materials) using two distinct scales: (1) Secure flourish measure: a scale consisting of two questions or items from each of five domains (happiness and life satisfaction, mental and physical health, purpose in life, character and virtue, and positive social relationships) (30, 31). (2) Beck Depression Inventory (BDI-II): an instrument screening and tracking of depression symptoms consisting of 21 items, rating on a 4-point scale ranging from 0 to 3 according to symptom severity (32). Beyond the questionnaires, each participant will answer one question about their overall health assessment.

Food frequency questionnaire (see SOP 11 in Supplementary materials): This semi-quantitative food frequency questionnaire (FFQ) is designed to explore the frequency of dietary consumption during the last year (long-term average diet). The FFQ included several food items, and it was adapted to vegan consumers, considering previously validated FFQs (33–36).

### 2.9 Sample size considerations

Correlations in nutritional epidemiology validation studies are usually in the range of 0.3–0.7 (31). Defining H0 as r < 0.3, to have 80% power to detect significant correlation at alpha level < 0.05, at least 85 vegan subjects are needed per group for final analysis (32). Taking into account potential dropouts or incomplete data and subgroup analyses (e.g., by sex, age group, etc.) we aim to enroll 50–100 vegan participants per study site, i.e., the recruitment will stop when 400 vegan and 170 omnivore subjects are available for final analysis. This would allow not only for sufficient power at the multinational level but would also allow for comparisons across countries for selected analyses.

The purpose of this manuscript is to describe a data collection protocol with a short overview of the planned analysis. Therefore, it does not include information on choice of statistical tests.

### 2.10 Statistical analysis

Baseline and demographic characteristics will be summarized using standard descriptive statistical summaries (e.g., means and standard deviations for continuous variables such as age and percentages for categorical variables such as gender).

Validation analyses of the screener will include correlating the total score with the data on nutrients, food groups, and total diet quality index from diet records adjusted for within-person variation using post-hoc statistical methods developed by Rosner (37). Diet records data will be triangulated with biomarker data and the screener data for further analysis (38).

Construct validity will be assessed by testing whether the measure relates as it should to other measures (e.g., age, gender, education) and by examining whether the total score calculated from the screener allows for sufficient variation among individuals. Concurrent/relative validity will be assessed by evaluating associations and agreement between the values obtained from the screener with selected values obtained from ‘gold standards’: diet records, concentration and recovery biomarkers, multi-metabolite signatures. Convergent validity will be assessed by examining whether the total score calculated from the screener allows for sufficient variation among individuals that are expected to have different levels of diet quality (e.g., age, gender, education level). We will also evaluate associations with biomarkers of disease risk, anthropometry, and demographic and lifestyle characteristics, as well as differences between vegans and omnivores. Finally, we will explore the internal consistency of the score by examining relationships among the index components.

We will evaluate correlations between vegan diet quality estimated by the vegan diet quality score calculated from diet records with dietary patterns derived from *a posteriori* dietary pattern analysis, and with biomarkers (both with ‘traditional biomarkers’) and with metabolic signatures. Empirically derived (or exploratory) dietary patterns will be derived using principal component analysis (PCA) and confirmatory factor analysis (CFA) (suitable for small samples) methods.

Our strategy involves the integration of NMR and LC–MS metabolomics to capture a comprehensive spectrum of metabolites in serum and establish correlations with data extracted from diet records and other biochemical analyses. To this end, we will exploit the data from omnivore groups as a control. This analysis will facilitate the development of an algorithm for assessing nutritional status based solely on dietary information. To achieve this goal, we will employ multivariable statistics and a machine-learning approach known as logistic regression with L1 penalization. These findings will contribute to the development of algorithms that enable precise self-assessment and tailored dietary recommendations.

### 2.11 Ethical consideration

The clinical study will be conducted in accordance with good clinical practice and the declaration of Helsinki (39). The study protocol was approved by the respective institutional review boards (see below).

Informed consent will be obtained before enrolment for the study in each subject. The investigator will make sure that subjects comprehend the nature of the study, the study procedures, and the risks and benefits of participation. There will be no penalty if a participant decides to withdraw from the study before it ends.

Study progress, data integrity, and ethical and safety concerns will be reviewed by the investigative team monthly (and more frequently if needed). Existing security provisions in accordance with GDPR and University security rules for the protection of personal identifying information are maintained. These measures include training of personnel, control of access to office space, and electronic security provisions. Data is stored on encrypted servers maintained by the Medical University of Vienna (MUW).

### 2.12 Storage of biological samples

Additional anonymized whole blood, serum and spot urine samples will be collected for long-term storage and reuse, and stored in the Medical University of Vienna Biobank. An additional consent for this purpose will be obtained from the participants. This form is optional and choosing not to donate biological samples for further use will not hinder participants from participating in the study.

## 3 Discussion

Plant-based diets, which exclude or limit the intake of animal products, are recognized as a more sustainable dietary pattern since they use fewer natural resources, reducing the environmental footprint (40). Notably, the adoption of nutritionally sound diets with a low environmental impact has gained considerable attention as a pivotal strategy in mitigating climate change (41) and promoting overall health (42). The transition of an important part of the European population toward plant-derived sources of food poses a need for public health care to secure adequate measures for prevention and risk-mitigation of nutritional deficiencies, namely for individuals who have adopted mostly plant-based dietary patterns, particularly in the vegan population.

Numerous studies have investigated the impact of a vegan diet on reducing the risk of diseases such as type 2 diabetes (11) and metabolic syndrome (43), improvement in the body weight and cardiometabolic risk factors (44), and potential benefits to gut microbiota (45).

Strict monitoring of the quality of vegan diet is essential to maximize its benefits and avoid nutritional deficiencies. Existing diet quality scores and tools for assessing nutrient intake used in cohort studies like NutriNet Sante, Nurses’ Health Study, and UK Biobank (46–48), as well as diet quality indices like the Healthy Eating Index-2015 (49) developed for omnivorous populations, are not suitable for application to vegan populations. These tools predominantly incorporate components based on animal-derived foods, such as red meat and dairy, while lacking essential components of vegan diets, including alternative protein sources like soy products, plant-based milk, dairy substitutes, and novel vegan foods.

There is an ongoing effort to make more precise and valid diet quality scores and screening tools specifically for vegans, one example is the Diet Quality Score for Vegans (DQS-V) that offers a comprehensive metric for evaluating diet quality among adult vegans based on the Swiss dietary recommendations for vegans (50).

The development and validation of a diet screener for vegans has important implications for healthcare professionals, public health initiatives, and environmental sustainability. The clinical study and its subsequent findings will enhance our understanding of diet quality and nutritional status assessment in populations that predominantly reduce their consumption of animal products. Moreover, untargeted metabolomics emerges as a valuable instrument for detecting metabolic changes linked to diet quality (51). A wide range of metabolites in biospecimens in the context of detailed dietary intake data would allow the development of an algorithm to assess nutritional status based on dietary intake data alone (52). These findings will contribute to the development of algorithms that allow for accurate self-assessment and refined, personalized dietary recommendations (53).

The final output, the validated VEGANScreener tool, will allow for a quick assessment of nutritional adequacy and potential risks of vegan diets. The findings of the ongoing evaluation study, along with the VEGANScreener itself, will be made accessible to a broad audience encompassing public health professionals, primary healthcare providers, dietitians, nutritionists, stakeholders, food producers, and other end-users beyond the vegan community. The tool would be translated to end-user products, such as web-based applications, that could be used both for further research purposes as well as fieldwork among medical professionals. As such, the tool will have the potential to address and mitigate safety concerns related to the global dietary shift toward plant-based sources, particularly in the public health sector. By providing a validated tool for assessing vegan diets, this tool can contribute to the reduction of emissions from food production and the promotion of sustainable dietary patterns.
