# Supplementary material for: Development and evaluation of a web-based diet quality screener for vegans (VEGANScreener): a cross-sectional, observational, multicenter, clinical study
Source: Front Nutr. 2024 Oct 22;11:1438740. doi: 10.3389/fnut.2024.1438740 (PMC11534613; doi:10.3389/fnut.2024.1438740)
Supplement: Supplementary file 1 [file Data_Sheet_1.docx]

**SOP 1 Eligibility questionnaire**

Dear potential participant, we are very pleased that you are interested in the VEGANScreener study that the main objective is the prevention and early detection of nutritional deficiencies in the diet of European individuals who follow a vegan diet.

This questionnaire will take approximately **5-10 minutes** and aims to **verify if you meet the inclusion criteria of the study**. If you do, VeganScreener personnel will contact you. Please answer all questions completely.

Your participation in the study is **voluntary**. You can terminate your participation at any time without giving reasons and without any disadvantages. You will not be asked to provide any identifying information until we have verified your eligibility in the study. At the end of the screening questionnaire, the website will let you know if you are conditionally eligible. Only in case you are conditionally eligible, you will be asked to enter your name, contact information, and some additional information in order for one of our research assistants to contact you.

The collected data will be stored and is subject to legal **data protection regulations**. It is guaranteed that information will be handled confidentially, only used for the purposes of the VEGANScreener study and will not be passed on to uninvolved third parties. The evaluation of the data is exclusively anonymous. If the results are published in a scientific journal, the data will not reveal who participated in the study.

If you have any **questions or uncertainties**, please contact our VEGANScreener team by e-mail at XXX@.com at any time.

We are pleased to be able to make an essential contribution to the scientific investigation of the vegan community with you.

With kind regards,

Your VEGANScreener study team

1. Date: _ _ /_ _ /_ _ _ _ (day) (month) (year)

2. Sex: Men Women Diverse

3. Age: _______**(<18 or >65 years old, EXCLUDED)**

*Only for women and diverse:

4. Are you currently pregnant or suspect you might be pregnant? **(if the answer is yes, EXCLUDED)***

Yes ___ No ___

5. Are you currently breastfeeding? **(if the answer is yes, EXCLUDED)***

Yes ___ No ___

6. Weight (kg): ______

7. Height (m): ________

8. (Automatic calculation, hidden from participant) BMI (kg/m^2^): ___________ **(>30 kg/m^2^, EXCLUDED)**

9. Have you ever been diagnosed by a doctor with these diseases? **(If the answer is yes, EXCLUDED)**

|  | **Yes** | **No** |
| --- | --- | --- |
| Diabetes type 1 |  |  |
| Diabetes type 2 |  |  |
| Gestational diabetes |  |  |
| Secondary diabetes |  |  |
| Pre-diabetes |  |  |
| Thyroid disease |  |  |
| Cancer |  |  |
| Inflammatory bowel disease |  |  |
| Chronic pancreatitis |  |  |
| Other malabsorption |  |  |

Other malabsorption, please specify:__________________ (If **answer to “other malabsorption” is “yes”**)

10. Which of the following definitions most closely describe your eating behavior over the past two years (select one answer)?:

1. Vegan (you strictly follow a plant-based diet only, i.e. no consumption of meat, fish, dairy or eggs) **(go to questions 11-16)**
2. Vegetarian (you do not eat meat at all, but you consume milk and dairy products, eggs, fish..) **(EXCLUDED)**
3. Reductarian/ flexitarian (you eat mainly foods of plant origin, but allow yourself to sometimes, on rare occasions, eat some meat, fish, eggs or dairy) **(EXCLUDED)**
4. Omnivore (your diet includes meat, fish, eggs, milk and/or other food of animal origin with no particular restrictions) **(go to questions 17-18)**

**FOR VEGANS ONLY**

11. How long have you been a vegan? Do not include any periods of vegetarian or flexitarian or reductarian diet.

_____/_____ (years/months)

(**for vegans/raw vegans ≥2 years, if not EXCLUDED)**

12. When did you start your current vegan diet? Date: __ __ __ __ (year)

Warning: The information provided on start and duration of the current vegan diet do not match! **(If answers to questions 11 and 12 do not match).**

13. Were there occasions **during the last two years** when you exceptionally consumed meat and/or meat products (e.g., sausage)? (**If the answer is 3 or 4, EXCLUDED**)

1. Never
2. Less than 1 time per month
3. 1-2 times per month
4. ≥3 times per month

14. Were there occasions **during the last two years** when you exceptionally consumed fish/fish products? (**If the answer is 3 or 4, EXCLUDED**)

1. Never
2. Less than 1 time per month
3. 1-2 times per month
4. ≥3 times per month

15. Were there occasions **during the last two years** when you exceptionally consumed milk/dairy products? (**If the answer is 3 or 4, EXCLUDED**)

1. Never
2. Less than 1 time per month
3. 1-2 times per month
4. ≥3 times per month

16. Were there occasions **during the last two years** when you exceptionally consumed egg/egg products (meaning also eggs contained in e.g., cakes, pasta or other ready-made products)? (**If the answer is 3 or 4, EXCLUDED**)

1. Never
2. Less than 1 time per month
3. 1-2 times per month

1. ≥3 times per month

**FOR OMNIVORES ONLY**

17. Have you been following this diet for more than two years?

Yes No **(if the answer is no, EXCLUDED)**

18. **During the last two years,** often did you consume meat and/or meat products (e.g., sausage)?* (**If the answer is 1-5, EXCLUDED**)

1. Never
2. Less than 1-3 times per month.
3. 1 time per week
4. 2 times per week
5. 3-4 times per week
6. 5-6 times per week **(INCLUDED)**
7. Daily (**INCLUDED)**
8. Several times a day **(INCLUDED)**

*This does not include plant-based meat alternatives

**INCLUDED/EXCLUDED (Automatic calculation)**

**If the participant does not meet all inclusion criteria as specified in questions 3-5, 8-11 and 13-18 -> Excluded**

**If the participant meets ALL inclusion criteria as specified in questions 3-5, 8-11 and 13-18 -> Included**

**GROUP (Automatic calculation hidden from participant, based on question 10)**

Vegan Control

First name: ___________________

Last name: __________________________

Date of birth: _ _/ _ _ / _ _ _ _ (day) (month) (year)

E-mail: _____________________________

Please re-enter your E-mail:_________________

**If e-mails do not match:**

Warning: *E-mail addresses do not match!*

Phone number: ______________________

Zip code:_________________

**SOP 2 Personal Medical History Questionnaire**

**CARDIOVASCULAR HISTORY**

1. Coronary artery disease (myocardial infarction, angina, congestive heart failure):

Yes

No

If answer is “yes”: Year of diagnosis:______



1. Limb ischemia:

Yes

No

If answer is “yes”: Year of diagnosis:______



1. Stroke:

Yes

No

If answer is “yes”: Year of diagnosis:______

1. Hyperlipidemia:

Yes

No

If answer is “yes”: Year of diagnosis:______

1. Hypertension:

Yes

No

If answer is “yes”: Year of diagnosis:______

1. Pulmonary embolism/deep vein thrombosis:

Yes

No

If answer is “yes”: Year of diagnosis:______

**METABOLIC AND ENDOCRINOLOGICAL DISORDERS**

1. Diabetes (type 1, type 2, gestational, secondary, prediabetes):

Yes

No

If answer is “yes”: Year of diagnosis:______

1. Hypothyroidism (autoimmune, other):

Yes

No

If answer is “yes”: Year of diagnosis:______

1. Hyperthyroidism (autoimmune, other):

Yes

No

If answer is “yes”: Year of diagnosis:______

1. Goiter:

Yes

No

If answer is “yes”: Year of diagnosis:______

1. Other endocrinological disorder:

Yes

No

If answer is “yes”:

Namely:_____

Year of diagnosis:______

**IRON METABOLISM**

1. Anaemia (sideropenic, other):

Yes

No

If answer is “yes”: Year of diagnosis:______

1. Sideropenia:

Yes

No

If answer is “yes”: Year of diagnosis:______

**BONE HEALTH**

1. Vitamin D deficiency:

Yes

No

If answer is “yes”: Year of diagnosis:______

1. Osteoporosis or osteopenia:

Yes

No

If answer is “yes”:

Localization___________________

At the age of:______



1. Fractures:

Yes

No

If answer is “yes”:

Localization___________________

At the age of:______

**PAST SURGICAL HISTORY**

1. Surgery:

Yes

No

If answer is “yes”:

Type of surgery:________

Year:______

**OTHER CHRONIC DISEASE**

1. Pulmonary:

Yes

No

If answer is “yes”: Year of diagnosis:______

1. Rheumatological:

Yes

No

If answer is “yes”: Year of diagnosis:______

1. Nephrological:

Yes

No

If answer is “yes”: Year of diagnosis:______

1. Hepatological:



Yes

No

If answer is “yes”: Year of diagnosis:______

1. Gastrointestinal:

Yes

No

If answer is “yes”: Year of diagnosis:______



1. Neurological:

Yes

No

If answer is “yes”: Year of diagnosis:______

1. Urological:

Yes

No

If answer is “yes”: Year of diagnosis:______



1. Psychiatric:

Yes

No

If answer is “yes”: Year of diagnosis:______

1. Oncological:

Yes

No

If answer is “yes”: Year of diagnosis:______



1. Gynaecological:

Yes

No

If answer is “yes”: Year of diagnosis:______



1. Hematological:

Yes

No

If answer is “yes”: Year of diagnosis:______

1. Other:

Yes

No

If answer is “yes”:

Other, please specify:_______

Year of diagnosis:______

**ALLERGIES AND INTOLERANCES**

1. Food allergy:

Yes

No

If answer is “yes”:

Namely:_______

Year of diagnosis:______

1. Food intolerance:

Yes

No

If answer is “yes”:

Namely:_______

Year of diagnosis:______

1. Inhalation allergy (rhinoconjunctivitis, rhinosinusitis, bronchial asthma):

Yes

No

If answer is “yes”: Year of diagnosis:______



1. Atopic dermatitis:

Yes

No

If answer is “yes”: Year of diagnosis:______

1. Other:

Yes

No

If answer is “yes”:

Namely:_______

Year of diagnosis:______

**Current pharmacotherapy, please check all that apply:**

Beta blockers

Diuretics

ACE inhibitors

ACE-II inhibitors

Calcium channel blockers

Alpha sympatholytics

Central acting antihypertensive drugs

Antiaggregants

Anticoagulants

Hypolipidemic drugs

NSAIDs

Corticosteroids

Biological therapy

Hormonal replacement therapy

Antihormones

Hormonal contraception

Iron replacement therapy

Other

None of the above

If answer “other” is selected: Other, please specify:_______

**FAMILY HISTORY**

1. Has any of your first-degree relatives (father, mother, brother, sister) suffered from any of the following illnesses? Please check all that apply:

Heart disease

Hypertension

Hyperlipidemia

Stroke

Diabetes type 1

Diabetes type 2, secondary diabetes, gestational diabetes or prediabetes

Cancer

Obesity

None of the above

**OTHERS**

1. Your birth mode

Vaginal delivery



Caesarean section

Don’

t know

1. How long have you been breastfed? ________ months / I don’t know

**QUESTIONS FOR WOMEN ONLY**

**Gynaecological history**

1. Age at first period:________
2. Periods:

Regular

Irregular

Menopause

1. Age of menopause: ________
2. Which of the following best describes how your menopause began?

Natural menopause

Surgery

Radiation or chemotherapy

I don’t know



1. Pregnancies:_____________

1. Have you ever taken oral hormonal contraceptives?

Never



Previously



Currently

If answer is “previously “or “currently”: How many years?______



1. Have you ever taken hormonal replacement therapy?

Never



Previously



Currently

If answer is “previously “or “currently”: How many years?______

**SOP 3 Clinical Examination**

*Anthropometric measurement*

Height, weight, waist circumference, and hip circumference are collected by trained dietitians with the participants in light clothing and without shoes. Height will be measured with a calibrated wall-mounted stadiometer. Weight is measured, using a calibrated scale. Waist circumference is determined midway between the lowest rib and the iliac crest. Hip circumference is measured at the widest part. Waist and hip circumferences will be measured with the use of anthropometric tape.

*Blood pressure and heart rate measurement*

After resting for 5 minutes, while the participant is in a seated position, blood pressure will be measured in triplicate with a validated semi-automatic oscillometer and the average of three repeated measurements will be taken as the final blood pressure. Please, use the left arm, if possible.

**Clinical Examination Form**

Weight 1:______.____ kg Height 1:______.___ cm BMI:__________ kg/m^2^

Weight 2:______.____ kg Height 2:______.___ cm

Waist circumference 1:________.___ cm

Waist circumference 2:________.___ cm

Hip circumference 1:________.___ cm

Hip circumference 2:________.___ cm

Waist-hip ratio:_______.____

To measure blood pressure, please **use left arm** if possible.

**Measured arm:**  Left Right

Blood pressure 1: Systolic________ Diastolic _______ mmHg Heart rate:________

Blood pressure 2: Systolic________ Diastolic _______ mmHg Heart rate:________

Blood pressure 3: Systolic________ Diastolic _______ mmHg Heart rate:________

**SOP 4 Collection and Storage of Blood and Serum samples**

1. After an overnight fast (12 hours) venous blood in **total amount of 81,9 mL** will be sampled from antecubital vein:

**54,4 mL blood for dietary markers analysis:**

- - 3 x 2,7 ml EDTA monovettes, 2 stored right away at -20°C, 1 stored right away at -79 °C;
  - 1 x 2,7 ml EDTA monovette, centrifugated, supernatant aliquoted into 5 ml polypropylene tube, stored at -79 °C;
  - 1 x 2,7 ml EDTA/NaF monovette, centrifugated, supernatant aliquoted into 5ml polypropylene tube, stored at -20 °C;
  - 4 x 9 ml serum monovettes, centrifugated, the supernatants aliquoted into 4x 5ml polypropylene tubes, 2 polypropylen tubes stored at -79 °C, 2 polypropylen tubes stored at -20 °C
  - 1 x 4,9 ml serum-gel monovette, centrifugated, the supernatant aliquoted into 5mL polypropylene tube and stored at -79 °C

**9,8 mL blood for metabolomic analysis:**

- 2 x 4,9 ml serum-gel monovette, centrifugated, aliquoted into 8x500 uL into 2 ml screw cap micro tubes, stored at -79 °C

**17,7 mL blood for biobanking:**

- 1 x 7,5 ml serum monovette, centrifugated, aliquoted into 7x400 uL into 2 ml screw cap micro tubes, stored at -79 °C
- 2 x 3,4 EDTA monovette, centrifugated, aliquoted into 7x400 uL into 2 ml screw cap micro tubes, stored at -79 °C
- 1 x 3,4 ml EDTA monovette, aliquoted into 7x400 uL into 2 ml screw cap micro tubes, stored at -79 °C

1. The samples will be kept on ice during the handling and transport.

**SOP 5 Collection and Storage of Saliva samples**

- Saliva samples will be collected into 15 mL sterile conical centrifuge tubes during the morning (visit1).
- The subject must not eat, drink, brush and/or floss their teeth or smoke 12 hours prior to the sampling.
- Participants will rinse out their mouths with water 15 min before collection.
- Whole unstimulated saliva will be collected passively deposited to the specimen tube. 5 ml of saliva will be sampled.
- The acquired samples will be immediately centrifuged at 10 000 x g for 15 minutes in a refrigerated centrifuge at 4 °C.
- Following centrifugation, it is important to transfer saliva into 2 mL sterile polypropylene screw cap micro tubes in aliquots immediately – 8 x 500 µl.
- The residue on the bottom will be kept for analysis of the 
  microbiome in the original tube (15 ml sterile conical tubes)
- Saliva samples and the residue after centrifugation are placed in a freezer and stored at -80 °C.
- The samples should be kept on ice during handling and transport to the local freezer.

**SOP 6 Collection and Storage of Urine Samples**

**Collection and storage of spot urine samples**

- Spot urine samples will be collected during the morning (visit 1).
- A clean-catch mid-stream urine sample will be collected.
- Participants will be given the supplies and instructions by the clinical staff about collecting a clean-catch mid-stream urine sample:
  - Go to the bathroom, wash your hands with soap and warm water, and dry your hands with a paper towel.
  - Remove the lip from the urine collection cup.
  - Void a small amount of urine into the toilet and stop mid-stream.
  - Void urine into collection cup until at least half full.
  - Replace the lid of the urine collection cup. Make sure it is screwed on tightly.
  - Place the urine collection cup into the plastic bag.
  - Wash hands with soap and warm water and dry hands.
- Urine will be aliquoted into 3 x 10 ml urine monovette and stored right away at -20 °C.

**Collection, aliquoting, and storage of 24-h urine samples**

- For the 24-hour urine collection, participants will be provided with chemically clean, properly labelled urine containers, written instructions and a form.
- Participants will receive verbal instructions on how to collect the 24-hour urine :
  - Upon arising in the morning, urinate into the toilet, emptying your bladder completely. Do not put this sample into the container. Note the exact time on the form provided by the study staff (e.g. 7:40).
  - You may fix a safety pin on your underclothes to help you to remember that the urine voided after this first-morning voiding should be collected in the container.
  - Collect all urine voided for the next 24 hours in the urine container. All urine passed during the 24-hour time period (day and night) must be saved. Urine passed during bowel movements must also be collected; pass urine before having a bowel movement.
  - Keep the collected urine in a cool place (preferably < 10 ̊C)
  - The following morning, void completely again (the first time after awakening), and add
    this sample to the storage container. Make sure a full 24-hour period has been covered: if the time of the discarded urine on the first day was 7:40 o’clock, then you should empty your bladder and add this specimen into the container for collection on the second day, also at 7:40 o’clock. This completes your 24-hour collection. Note the time of this last collection on the form. Also, note the time of any specimens you missed. Record also disease complaints, use of medicine, and supplement use on the diary scheme.
  - The 24-hour urine specimen should be taken to the study site (agreement made with study staff) as soon as possible, within 1 day.
- Upon submission of the urine sample, any deviations from the urine collection protocol (incomplete collection, etc.) and use of medication will be checked with the subject.
- The date of weighing, urine weight plus container will be noted. Urine net weight will be calculated afterward since we have the weight of the empty container.
- The urine collection will be homogenized. When submitting two containers, each container of urine will be rotated and inverted at least 10 times before pouring into an Erlenmeyer flask. If only one container is filled with urine, it is not required to pour the urine into the Erlenmeyer flask.
- 2 x 10 ml of the homogenized 24-hour urine specimen using a pipette will be aliquoted. Aliquots will be immediately placed in a freezer and stored at -80 °C. The leftover urine will be discarded in the toilet.

**SOP 7 Diet Record**

**Instructions for your diet record**

- Keep the diet record for **four** non-consecutive days (**comprising of three weekdays and one weekend day**) over the course of 14 days. Please do not record two or more days in a row, but rather take a break of one to two days before recording your next day.
- If you did not eat your typical diet due to sickness or fasting, please skip that day and record the next day when you are again eating **“as usual”** (while keeping 3 weekday+1 weekend combination). If in dilemma, contact the study centre for guidance.
- **Do NOT change** what you usually eat and drink during the days when you are completing this diet record (e.g. to make it easier to record). Instead, eat and drink as you normally do.
- Write down everything you consumed (**from midnight to midnight** for each day). Include late night food and beverages, such as drinks during a concert/snack when waking up at night). Include small bites eaten “on the go” (e.g., at work or takeaways), even a sip of juice or a candy in between.
- **When and where?** - always note at what time and what place you ate or drank something. You can round time up to a full hour, e.g. 13:00. Location can be “at home” or: at friends, at parents, restaurant, café, clubbing, concert, takeaway food, street food, etc.
- **What?** - provide as much detail as possible about the foods, beverages, and supplements and detailed product description (e.g., type, flavor, **fat** content, etc.). Also list any additives (e.g., "multivitamin juice + vitamin C 100 mg"). If you use **sweetener**, specify type (e.g. cane sugar, stevia…) and amount (e.g. in tea spoons/sachets/drops) used. IMPORTANT: label each food or beverage reported within a meal with a), b), c), d) and so on. See examples on pages 8 and 9.
- **Brand** - Include a brand name for any packaged product. IMPORTANT: label each food or beverage reported within a meal with a), b), c), d) and so on. See examples on pages 8 and 9.
- If you take **pictures of the packaging**, please make sure to photograph the brand name and type (front packaging), the ingredients list, and the nutrition facts.
- **Preparation** - Indicate whether and how you processed the food before eating it. You can note here, for example, if you have peeled fruit and vegetables or if you have boiled, baked or fried food. IMPORTANT: label each food or beverage reported within a meal with a), b), c), d) and so on. See examples on pages 8 and 9.
- **Amount** – Whenever possible (e.g. when preparing meals at home), **weigh** all the prepared food and drinks you plan to eat/drink with a digital scale and write down the weight of each item in grams under **amount served**. If you have not eaten/drunk everything, please weigh the **leftovers** and note this in the "leftovers" field (see also instructions on page 2). Alternatively, you can attach recipes or list the ingredients and record the portion consumed using the portion size pictures or household measures. In case you weighed the food before preparation, please make sure that you wrote this down (e.g., "noodles weighed uncooked"). IMPORTANT: label each food or beverage reported within a meal with a), b), c), d) and so on. See examples on pages 8 and 9.
- When it is not possible to use a scale (e.g., when eating away from home), indicate the quantity a seither as kitchen utensils (e.g. tablespoon, teaspoon, small/large glass/cup/mug, small/large bowl/plate, scoop/ladle) or record the portion consumed using the portion size pictures on pages 3 and 4. You may also attach a photo of the portion and indicate whether there were leftovers (take photos of your meal and your leftovers **from an angle of 45 degrees and at a 1-arm distance** from the object and take a picture of the menu). See examples of photos on pages 10 and 11.
- Some foods such as beverages are consumed over the course of the day. If, for example, you drink from a water bottle during the day, enter the total amount consumed at the end of the day and note "**during the day**" in the “when and where” field (paper form) or select: 00:00 if you are completing the form in MyCap.
- **Supplements** - Indicate if you take nutritional supplements, e.g., vitamin supplements. Please note the dosage, brand name and detailed product description. You may also attach a photo of the product.

**Weighed amount:** for a meal prepared at home that consists of several components (e.g., potatoes, vegetables, and meat/tofu), we recommend the following procedure:


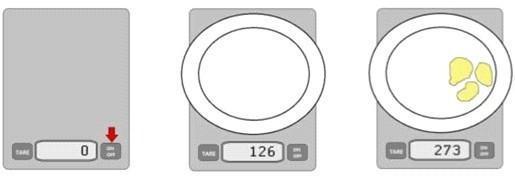


| 1) Place the scale on a level surface and turn it on. |  | 2)  Weigh the empty dishes and record the empty weight. |  | 3) Place the first component on the dish and record the total weight, indicating the component. |
| --- | --- | --- | --- | --- |


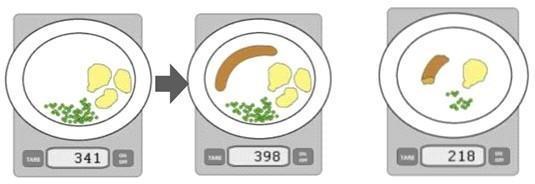


| 4) Now add the other components to the dishes one after the other and note the total weight added up for each component. Mark the addition in the report with a “+“ in front of each added component. |  | 5) After you finished eating the meal, if necessary, place the dishes with the leftovers on the scale and record the weight. |
| --- | --- | --- |


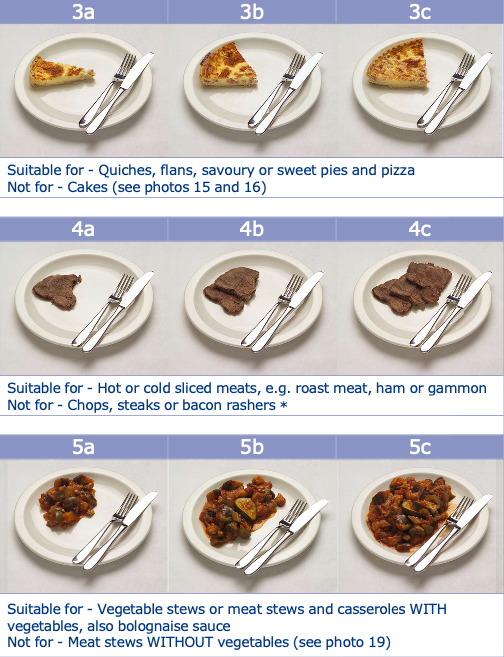


**Estimated amount:** For a meal eaten out, or a meal that was served to you and it is therefore not possible to weigh separate components, please use these pictures to determine the served amount. Source The EPIC-Norfolk Study (https://epic-norfolk.org.uk/wp-content/uploads/2020/10/Food_diary_v3.pdf). Used and adapted with permission.


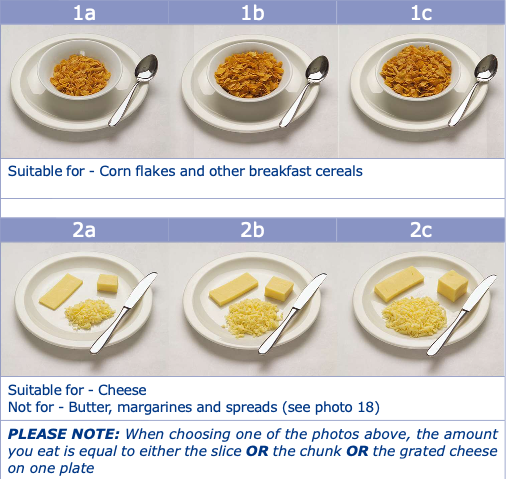


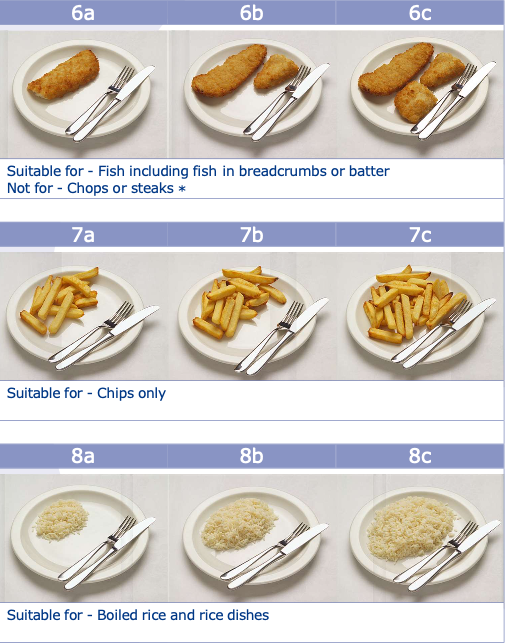


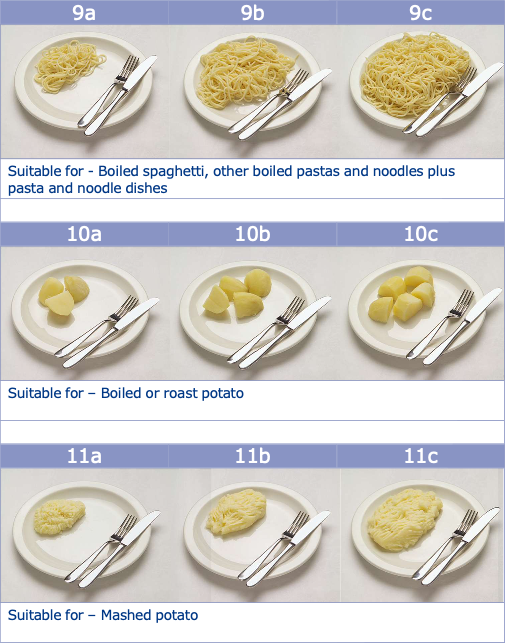


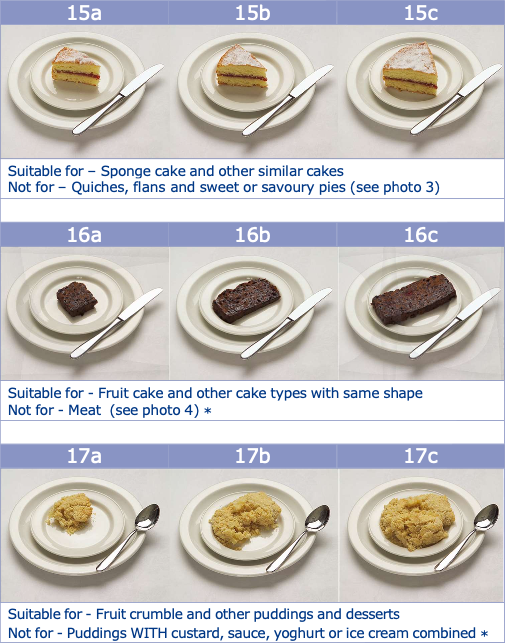


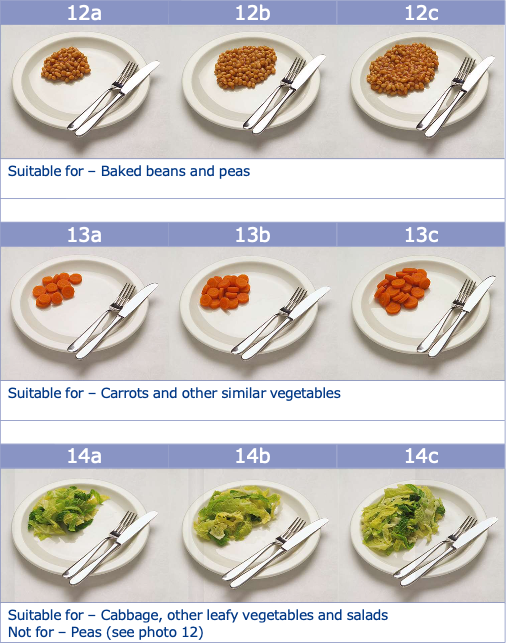


x


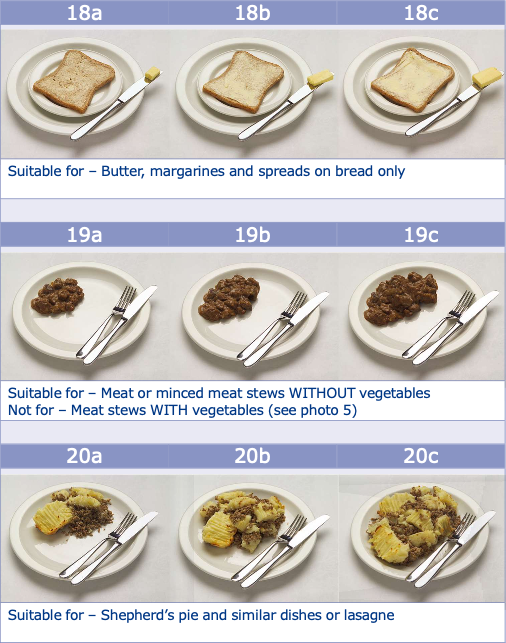


Study-ID:

For each diet record day, you will also be asked to answer the following three questions:

- Did you have any health complaints (e.g., vomiting, diarrhea, sore throat, fever) during this day of diet record?


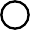
 yes
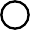
 no

If yes, briefly describe what health complaints you have had.

- Does this day of the diet record roughly correspond to your usual diet?


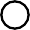
 yes
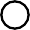
 no

If no, please briefly describe why and how diet deviated from usual during the days of the diet record (e.g., family celebration, more cake/sweets consumed).

- Please indicate all applicable properties of the table salt you used:


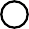
 I didn’t use salt


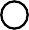
 Table salt (not-iodized)


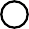
 Iodized table salt: Only iodized with fluoride with folic acid

*Thank you for your support!*

**Example for your diet record**

Study-ID: Day Date: Day of the week:

| **When and where?** | **What?**  **(food and drink described in detail)** | **Brand**  (except for fresh foods and beverages) | **Preparation** | **Amount served** [in g, ml, or household measures] | **Leftovers** [in g, ml, or household measures] |
| --- | --- | --- | --- | --- | --- |
| 7:00  At home | Bowl, empty |  |  | 113 g |  |
|  | a)+ oats, rolled, bio | a)dm |  | a)142 g |  |
|  | b)+ pear |  | b)Raw, peeled | b)199 g |  |
|  | c)+ oat milk | c)Oatly barista (see photo) |  | c)286 g |  |
|  | d)Bowl with leftovers |  |  |  | d)128 g |
|  | Cup, empty |  |  | 180 g |  |
|  | e)+ herbal tea “fennel” | e)Meßmer |  | e)315 g | e)0 g |
|  |  |  |  |  |  |
| 9:00  In café | a)Raw cake with a nut and date base, blue spirulina, apple, coconut cream, papaya, and strawberries |  |  | a)1 piece (see photo) OR (see picture 16b) | a)0 |
|  | b)Latte macchiato (oat milk with espresso) |  |  | b)Ca. 250 ml | b)0 ml |
|  | c)Sweetener (stevia) |  |  | c)1 sachet |  |
| 12:00  At parents |  |  |  |  |  |
|  | a)Pumpkin soup (see recipe) |  | a)Cooked | a)One big plate |  |
|  | b)Chickpeas (canned, cooked) |  |  | b)One bowl (see picture 12b) | b)0 g |
|  | c)Potato wedges |  | c)Oven-baked | c)4-5 small pieces (picture 10b) | c)0 g |
|  | d)Rapeseed oil |  |  | d)1 table spoon | d)0 g |
|  | e)Mineral water classic | e)Gerolsteiner |  | e)A big glass (about 250 ml) | e)nothing |
|  |  |  |  |  |  |
| 16:00  On the train | a)Banana |  | a)Peeled | a)One, medium sized | a)0 g |
|  | b)Protein bar (Hazelnut + nougat, vegan) | b)Barebells (see photo) |  | b)55 g | b)0 g |
|  |  |  |  |  |  |
| 18:00  Restaurant | a)Vegan burger with white and sweet potato fries |  |  | a)Shared with a friend (half of the portion was eaten by me)  See photo (before) | a)See photo (only if there are leftovers) |
|  | b) Burger bun |  | b)Toasted | b)1 bun |  |
|  | c) vegan burger patty (made from black beans) |  | c)Pan-fried | c)1 patty |  |
|  | d) Vegan cheese |  | d)Melted | d)1 slice |  |
|  | e) tomato |  | e)Raw | e)2 slices |  |
|  | f) pickled cucumber |  | f)Raw | f)2 pieces |  |
|  | g) red onion |  | g)Raw | g)2 slices |  |
|  | h) lettuce |  | h)Raw | h)2 leaves |  |
|  | i) ketchup |  |  | i)1 table spoon |  |
|  | j) mustard |  |  | j)1 table spoon |  |
|  | k) White and sweet potato fries |  | k)Fried | k)1 bowl (see picture 7b) |  |
|  | l) Vegan mayonnaise |  |  | l)4 table spoon |  |
|  | m) White wine |  |  | m)200 ml |  |
|  |  |  |  |  |  |
| During the day | a)Mineral water classic | a)Gerolsteiner |  | a)1500 ml | a)0 |
|  |  |  |  |  |  |

Supplements taken today (e.g., vitamins, minerals):

| **Brand name** | **Nutrients contained** | **Dosage (mg/tsp/drops)** |
| --- | --- | --- |
| Vitamin B12 drops, Sunday natural | Vitamin B12 | 1 drop á 200 µg |
| Watson Nutrition proveg omega 3+ | Vitamin D  Vitamin E  EPA  DHA  (EPA + DHA) | 1 capsule á  25 µg/1000 IU  3 mg  Min. 75 mg  Min. 150 mg  (250 mg) |

Study-ID:

**Examples of photos taken for the diet record**

| **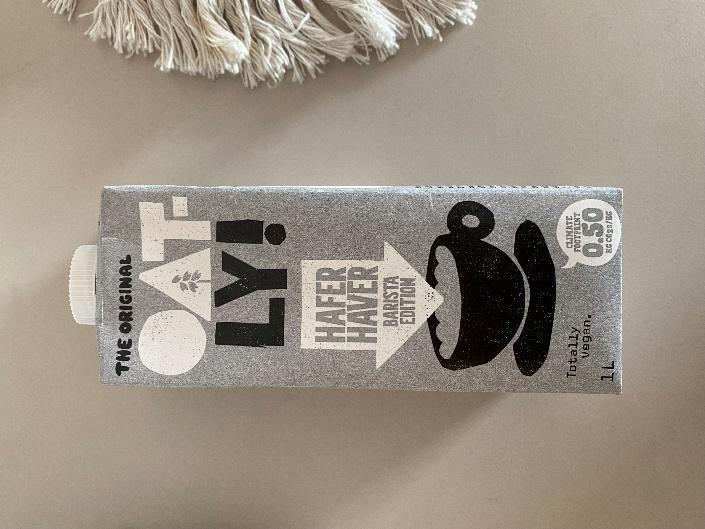** | **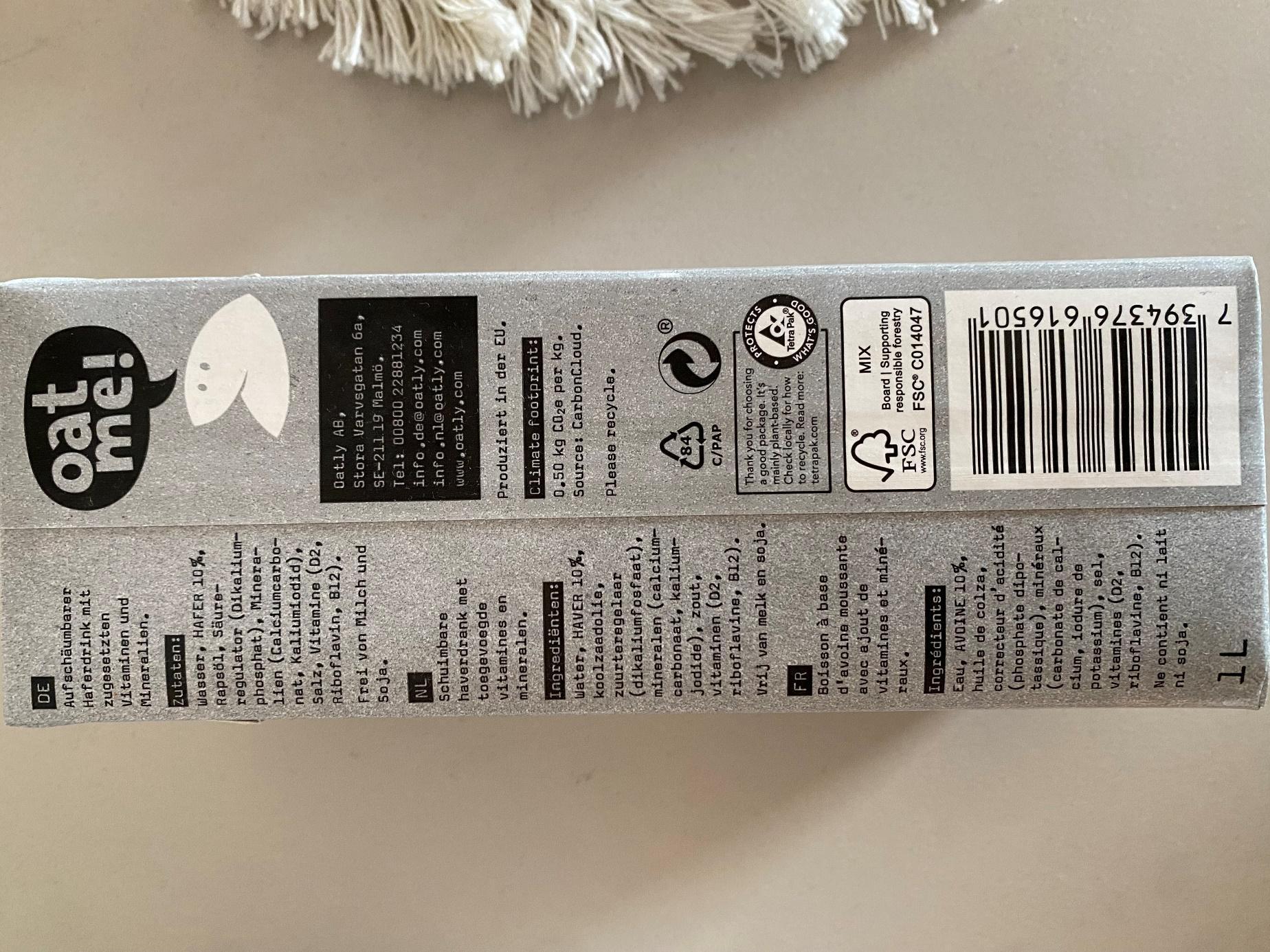** | **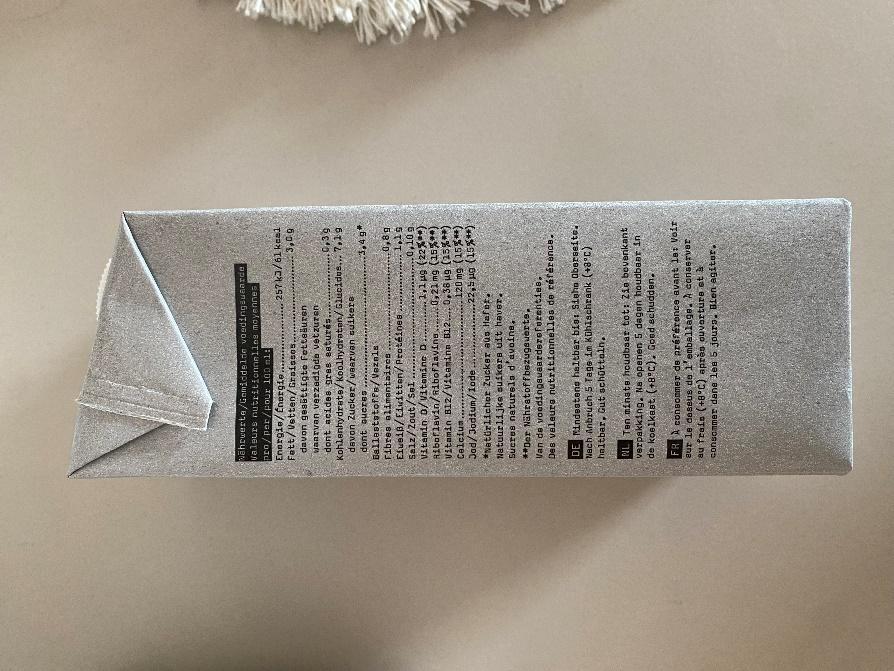** |
| --- | --- | --- |

| **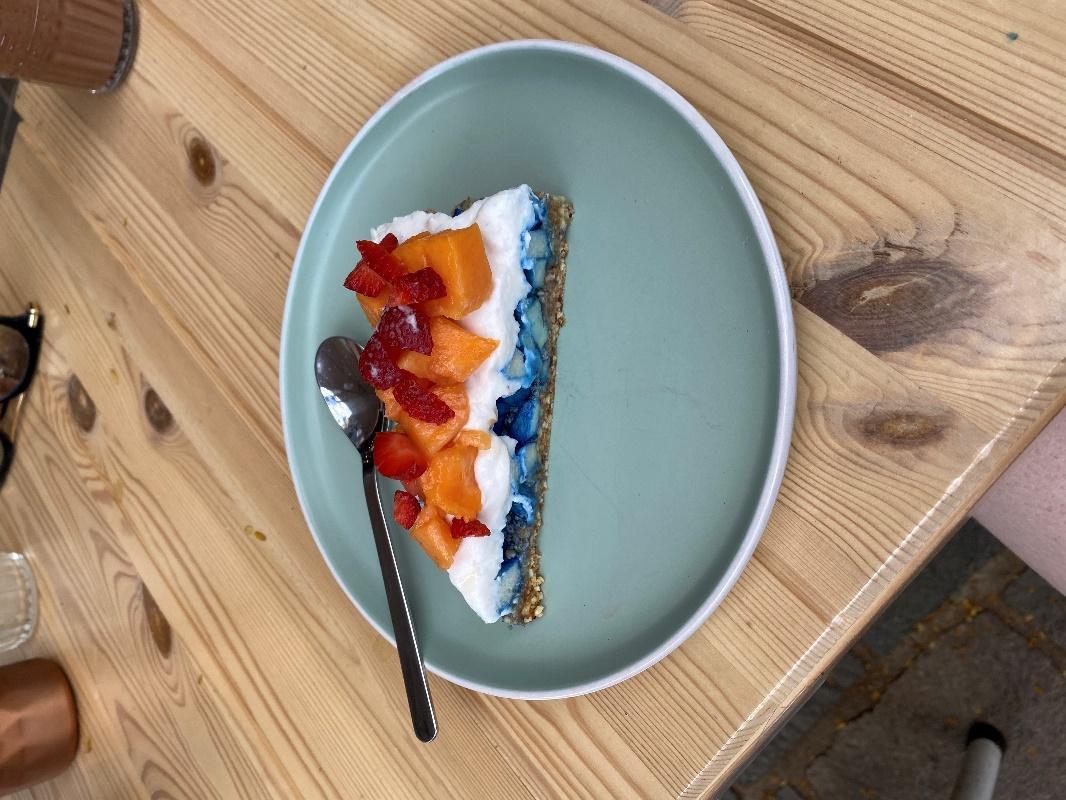** | **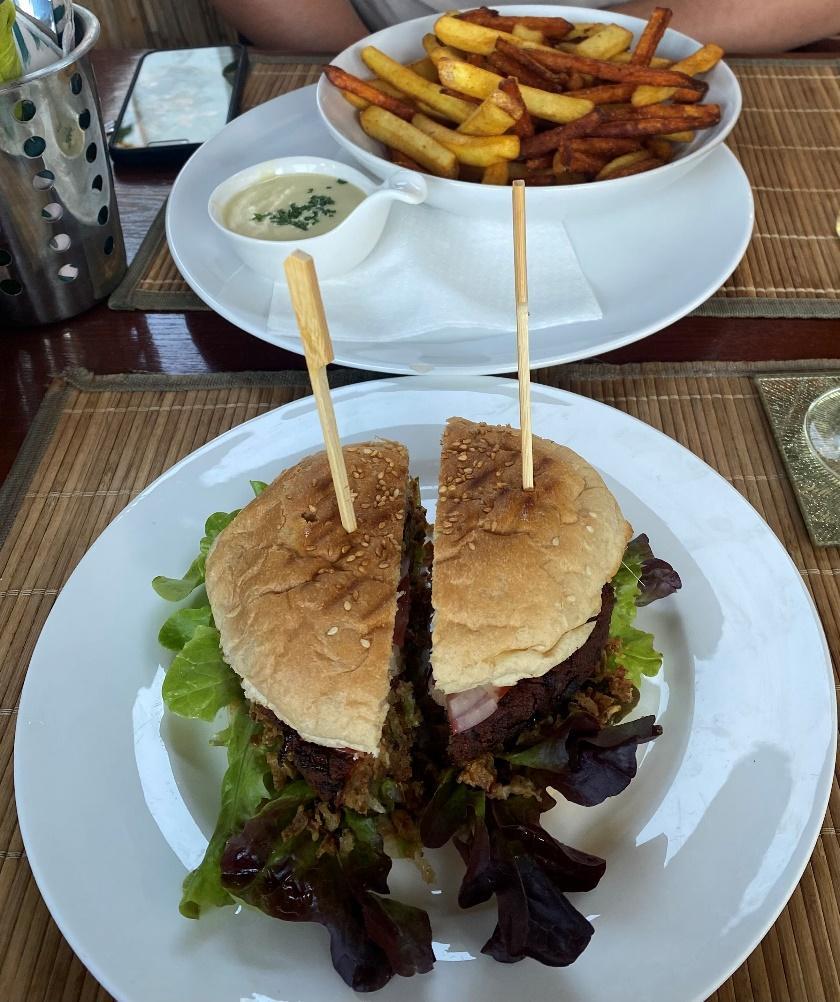** |
| --- | --- |

| **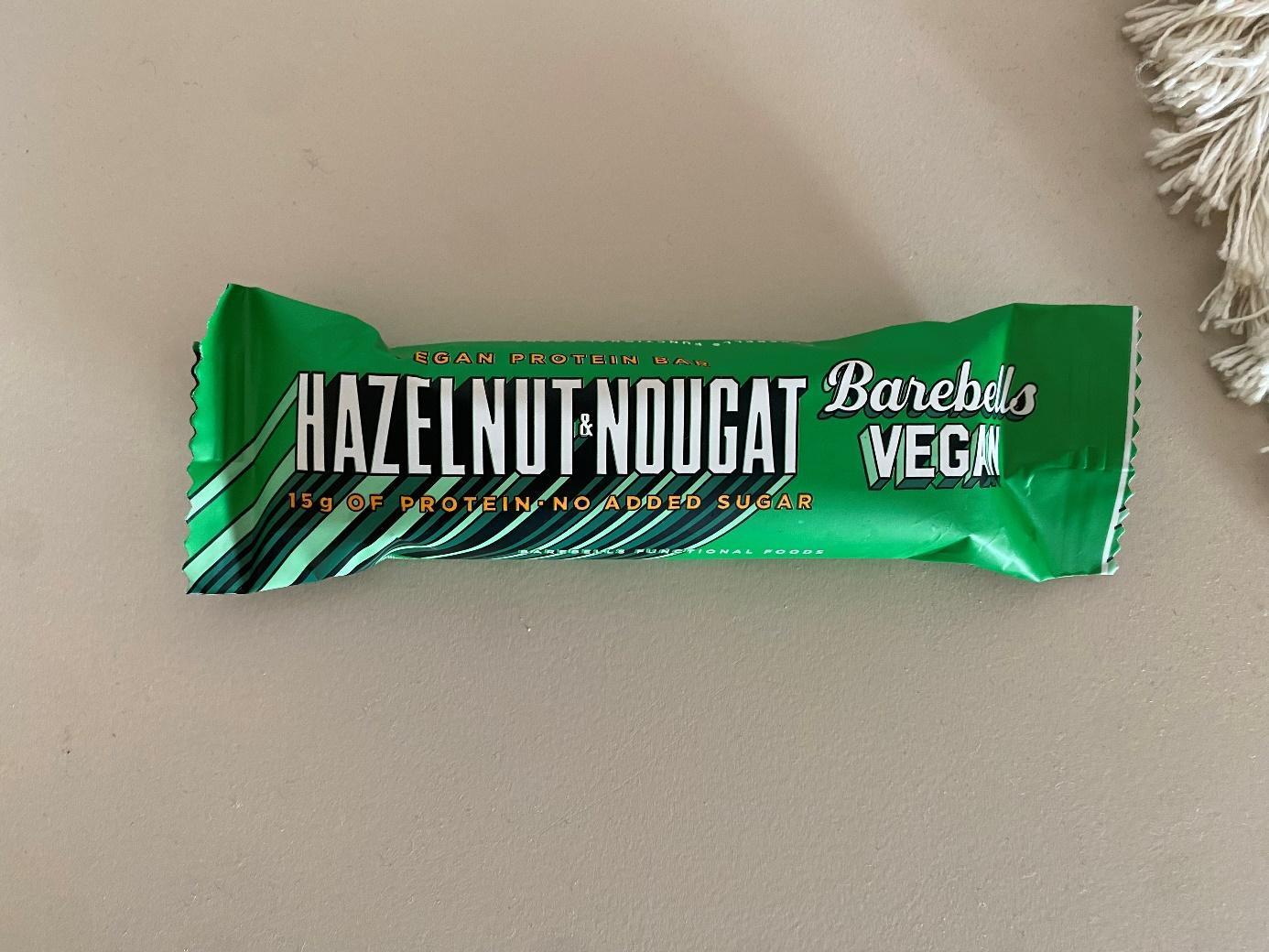** | **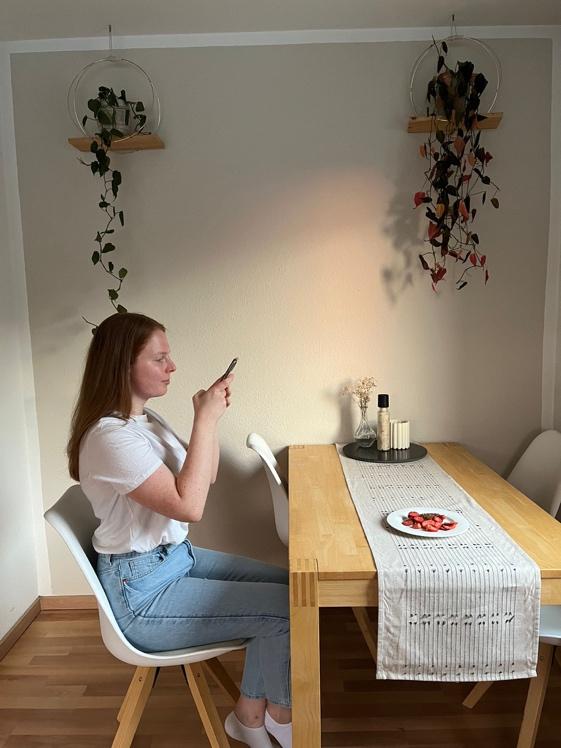** |
| --- | --- |
| **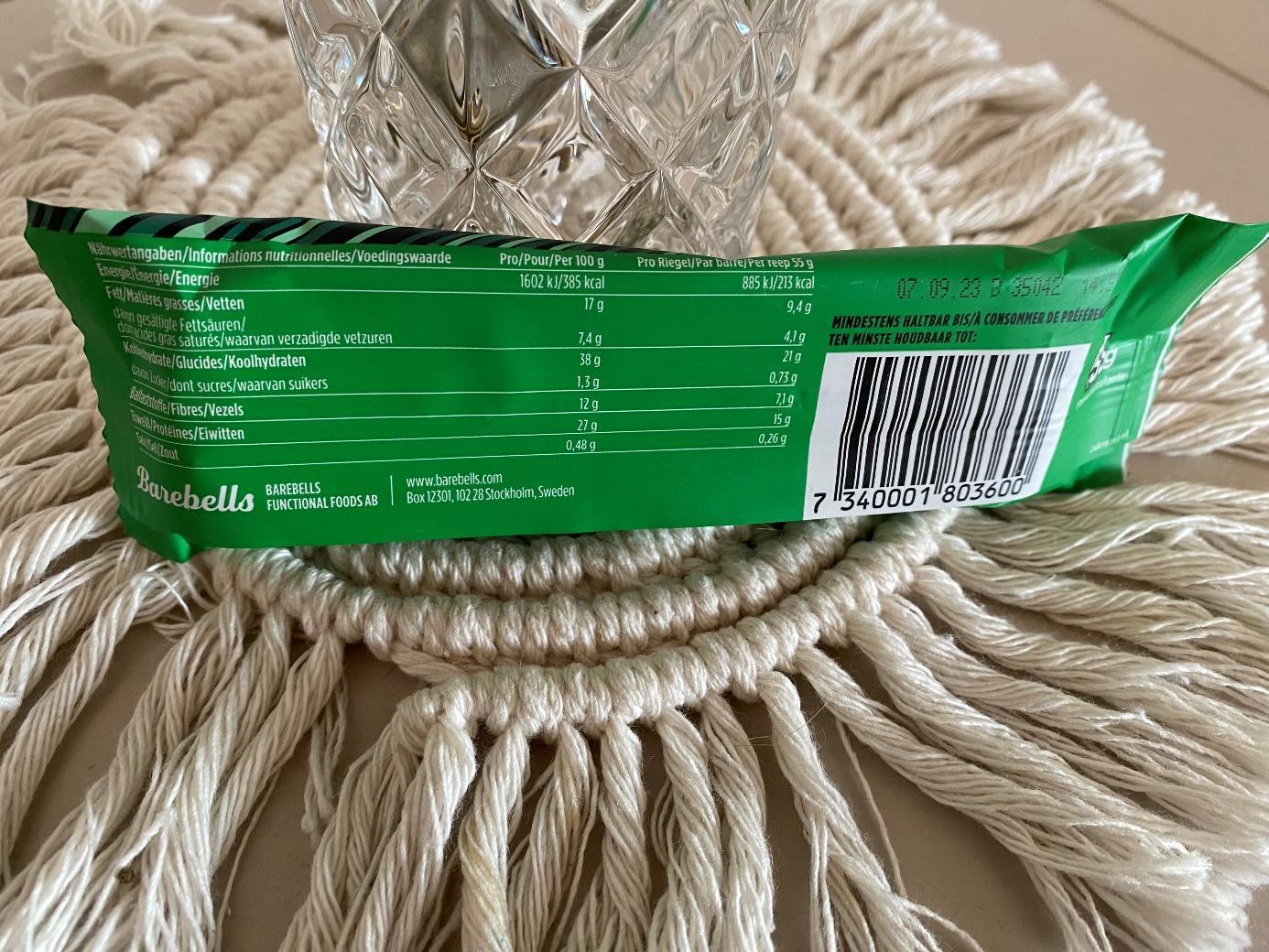** |  |
| **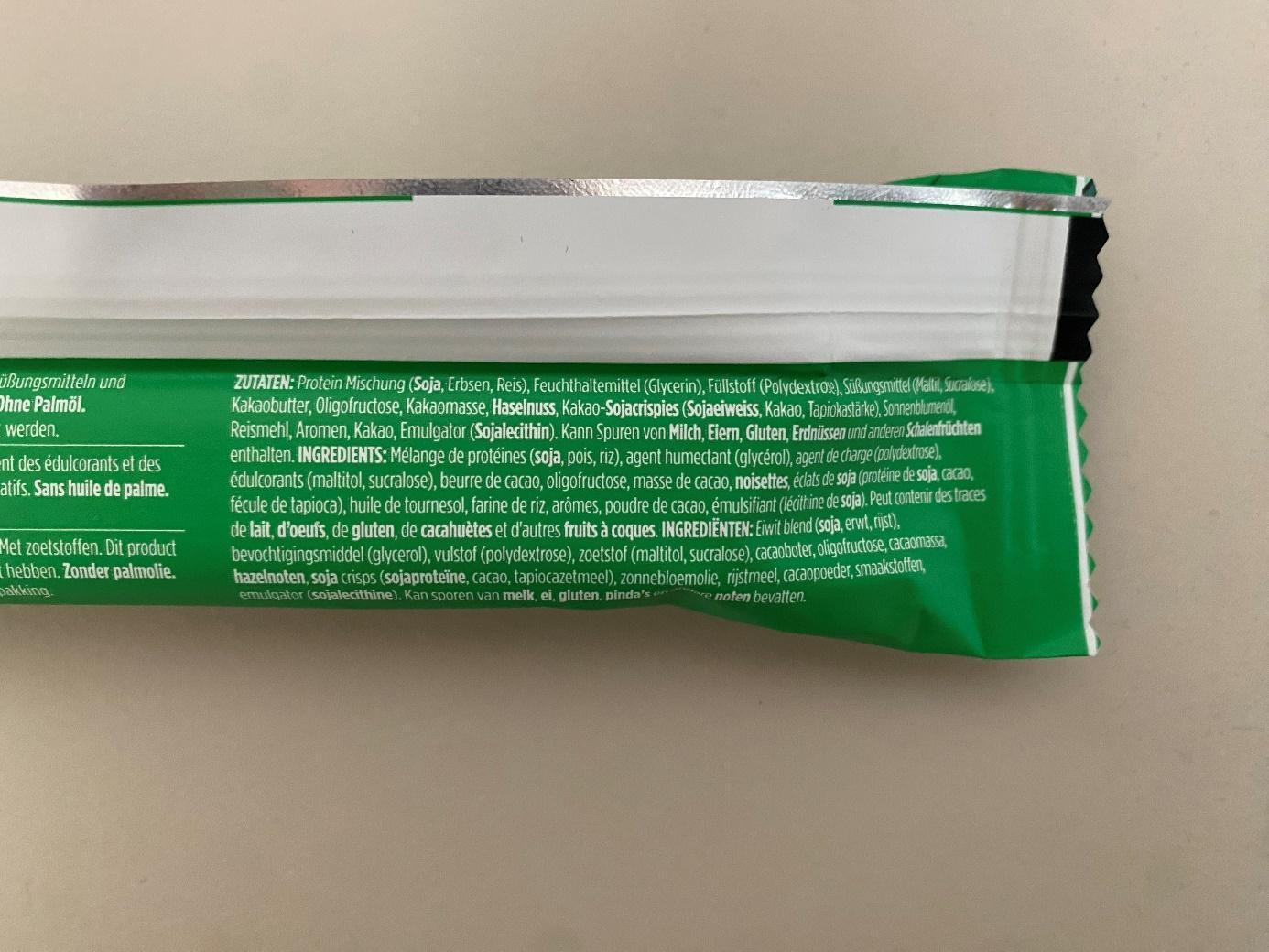** |  |

| **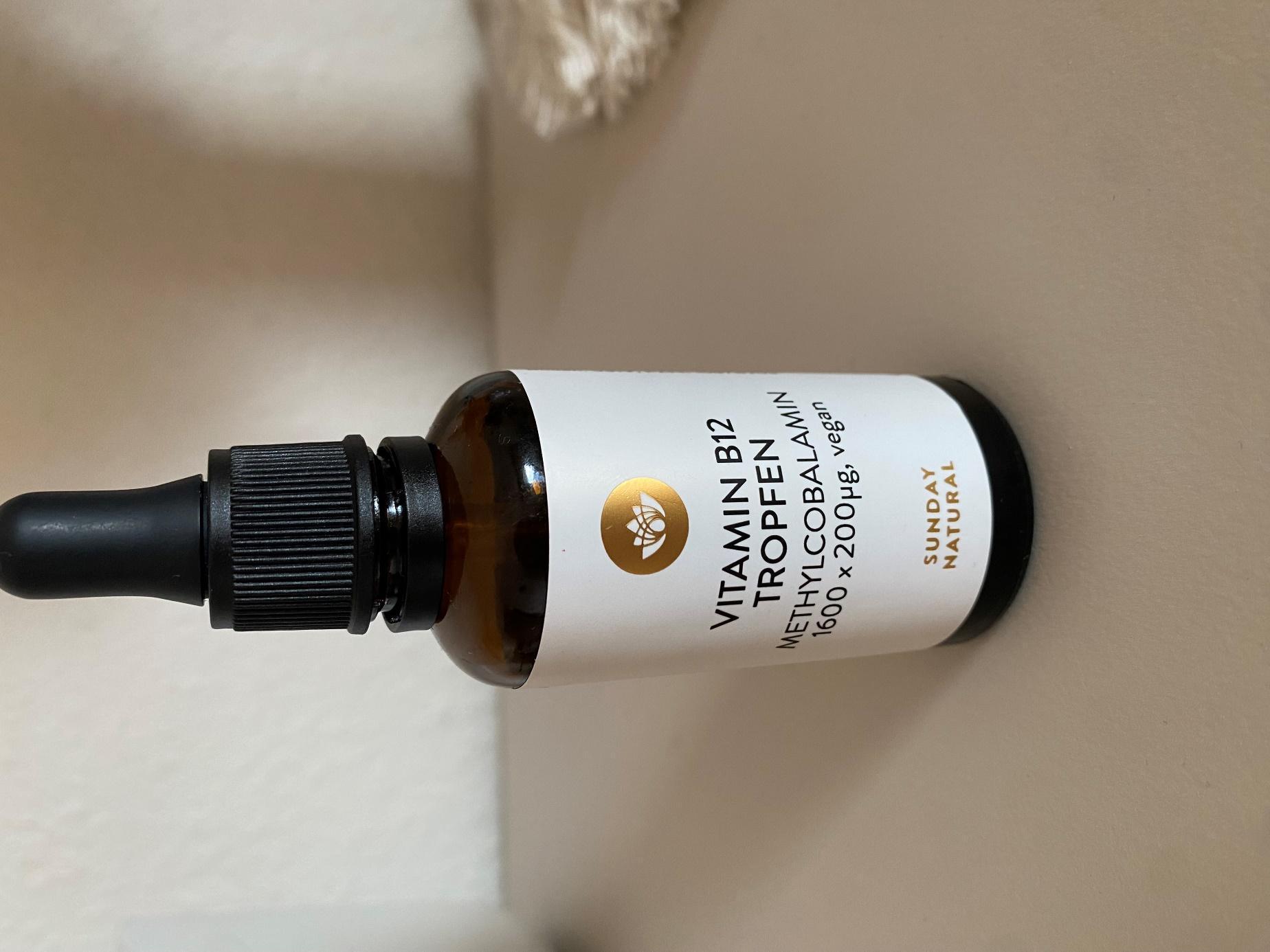** | **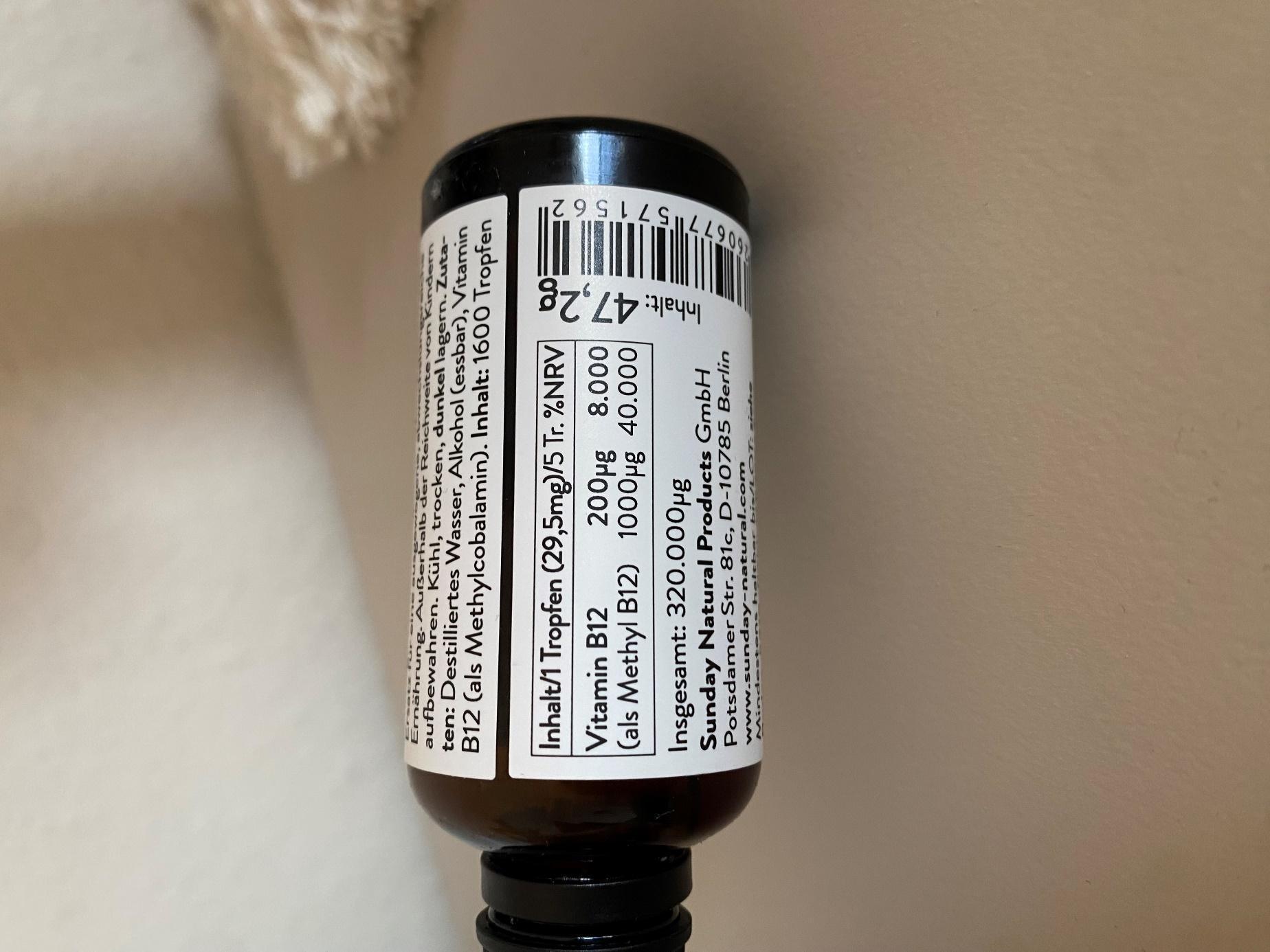** | **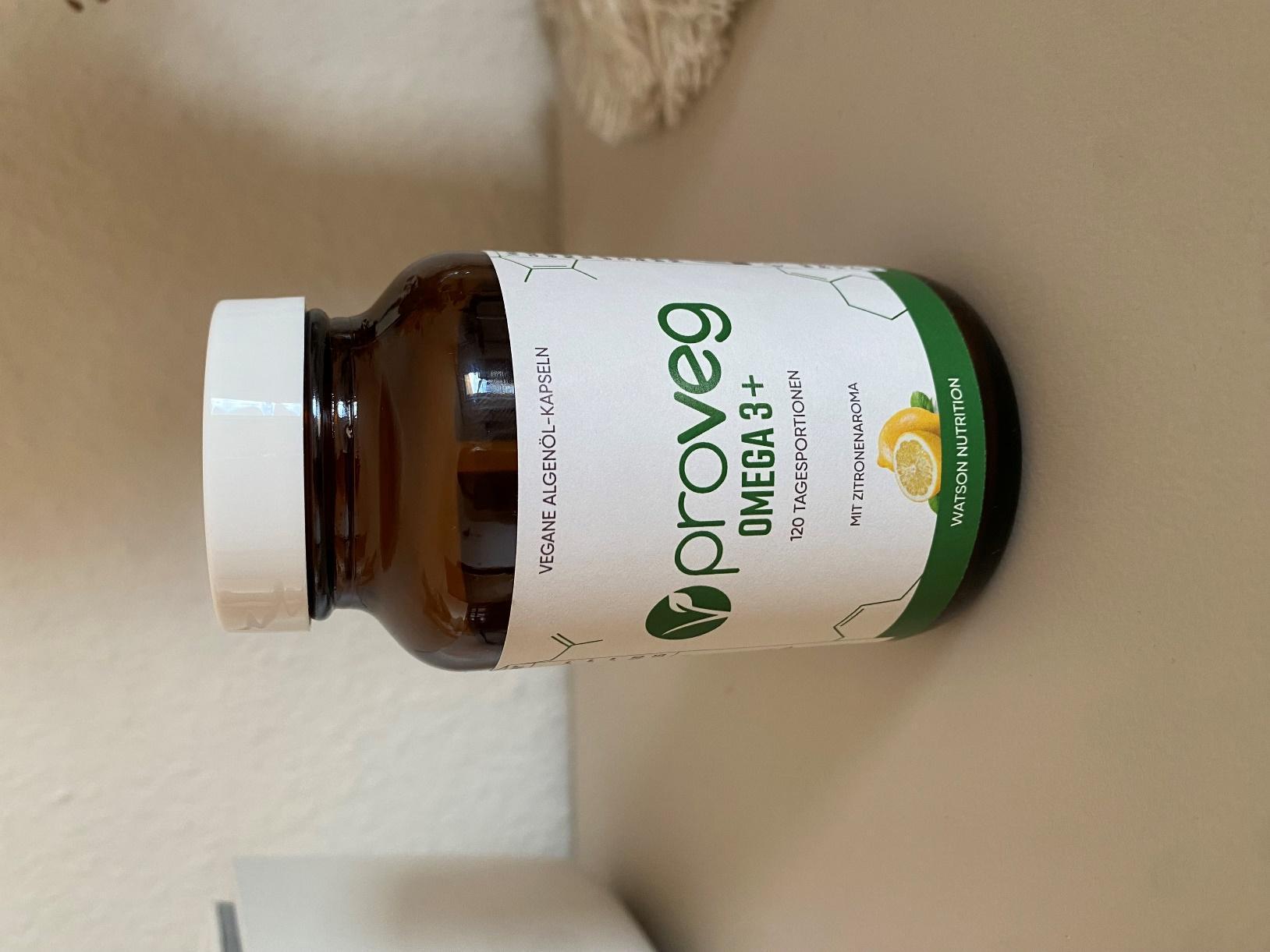** | **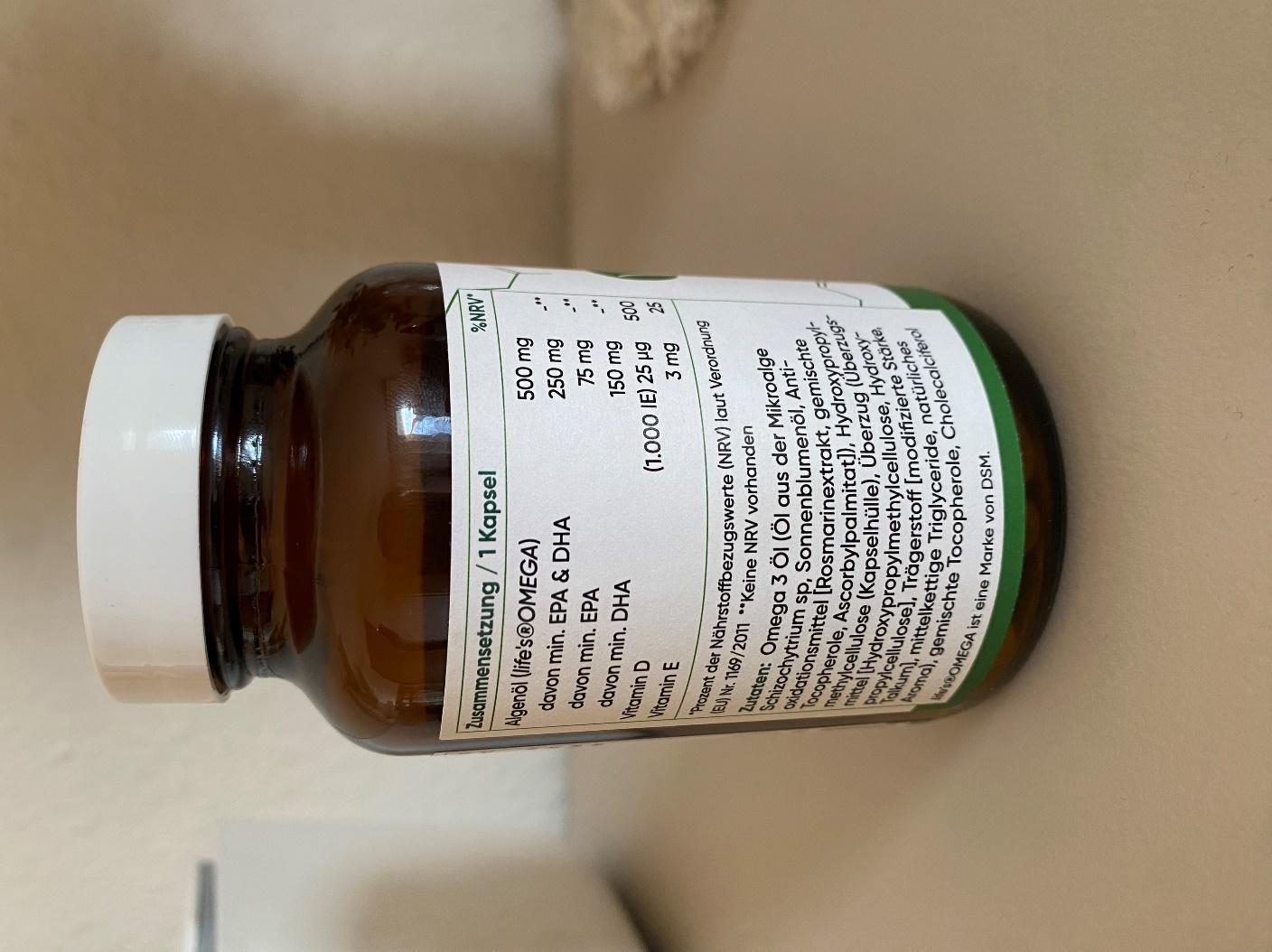** |
| --- | --- | --- | --- |

Study-ID:

Please enter here the recipes of the dishes you have eaten (the important thing is **not the individual recipe steps**, but the name of the food and the weight):

º

Pumpkin soup, day 1:

400 g Pumpkin (hokkaido)

45 g white onion

400 ml veggy stock (400ml tap water, one vegetable boulloin cube (Knorr))

Chili powder, black pepper, paprika powder

1 tbsp rapeseed oil

3 tbsp soy sauce

**Sending photos of your foods**

- If using MyCap: you will be prompted by the app to take photos when you enter information about meals. Any forgotten photos (e.g. of packaged foods): you can take them later and send us by email.
- If using paper form: name the files as: your last name_date_time. For example: Smith_20-3-23_15h.

**Submitting your completed diet records**

- If using MyCap: After completing a “task” in the App the 100% sign shows up. If there is an active internet connection the record is submitted automatically.
- If using paper form: send the first completed diet record with photos (day 1) immediately upon completion to the following email for checkup and feedback (xxx@xxx.xx), and then send all 4 completed diet records (i.e. photographs or scans of completed diet records to the same email immediately upon completion.
- Please write clearly.

**If you have any questions, please contact your study center:**

Contact person(s): xxx
By phone to: xxx, during the following days and hours: xxx.
By email: xxx@xxx.xx

Study center

Address

Postal code, city

Country

**Thank you for your participation!**

**Diet record paper form**

**
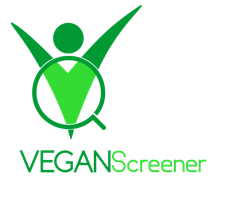
DIET RECORD**

Study-ID: Day Date: Day of the week:

| **When and where?** | **What?**  [food and drink described in detail] | **Brand**  [except for fresh foods and beverages] | **Preparation** | **Amount served**  [in g, ml, or household measures] | **Leftovers**  [in g, ml, or household measures] |
| --- | --- | --- | --- | --- | --- |
|  |  |  |  |  |  |
|  |  |  |  |  |  |
|  |  |  |  |  |  |
| **When and where?** | **What?**  [food and drink described in detail] | **Brand**  [except for fresh foods and beverages] | **Preparation** | **Amount served**  [in g, ml, or household measures] | **Leftovers**  [in g, ml, or household measures] |
|  |  |  |  |  |  |
|  |  |  |  |  |  |
|  |  |  |  |  |  |
|  |  |  |  |  |  |
|  |  |  |  |  |  |
|  |  |  |  |  |  |
| **When and where?** | **What?**  [food and drink described in detail] | **Brand**  [except for fresh foods and beverages] | **Preparation** | **Amount served**  [in g, ml, or household measures] | **Leftovers**  [in g, ml, or household measures] |
|  |  |  |  |  |  |
|  |  |  |  |  |  |
|  |  |  |  |  |  |
|  |  |  |  |  |  |
|  |  |  |  |  |  |
|  |  |  |  |  |  |

Supplements taken today (e.g., vitamins, minerals):

| **Brand name** | **Nutrients contained** | **Dosage (mg/tsp/drops)** |
| --- | --- | --- |
|  |  |  |
|  |  |  |
|  |  |  |
|  |  |  |
|  |  |  |
|  |  |  |
|  |  |  |
|  |  |  |
|  |  |  |
|  |  |  |
|  |  |  |
|  |  |  |
|  |  |  |
|  |  |  |
|  |  |  |
|  |  |  |
|  |  |  |

Study-ID: Day Date: Day of the week:

**RECIPES:**

1. Did you have any health complaints (e.g., vomiting, diarrhea, sore throat, fever) during this day?


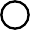
 yes
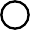
 no

If yes, briefly describe what health complaints you have had.

1. Does this day of diet record roughly correspond to your usual diet?

               
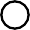
 yes
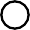
 no

If no, please briefly describe why and how diet deviated from usual during this day of the diet record (e.g., family celebration, more cake/sweets consumed).

1. Please indicate all applicable properties of the table salt you used:


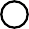
 I didn’t use salt


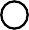
 Table salt (not-iodized)

               
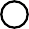
 Iodized table salt: Only iodized with fluoride with folic acid

**DO NOT WRITE BELOW (TO BE COMPLETED BY STUDY TEAM)**

Entry verified: yes/no

Notes/further action needed?:

SOP 8 VEGANScreener

| Over the past month, how often did you, on average, consume **at least one** serving of foods or beverages from the following food groups: | | | | | | **Never** | **Rarely/**  **1x/month** | **2-3x/**  **month** | **1x/**  **week** | **2-3x/**  **week** | **4-6x/**  **week** | **1x/**  **day** | **2x/**  **day** | **≥3 times**  **/day** | **1 serving example** |
| --- | --- | --- | --- | --- | --- | --- | --- | --- | --- | --- | --- | --- | --- | --- | --- |
| **Q1** | The following* **green** vegetables, fresh or cooked; whole, cut or blended: | | | | |  |  |  |  |  |  |  |  |  | *1 handful cooked /*  *2 handfuls fresh vegetables* |
|  | Broccoli  Kale  Bok choy  Arugula | | Chinese cabbage  Green cabbage  Savoy cabbage | | Brussel sprout  Endive Artichoke |  |  |  |  |  |  |  |  |  |  |
| **Q2** | The following* **dark orange and red** fruits and vegetables, fresh or cooked; whole, cut or blended: | | | | |  |  |  |  |  |  |  |  |  | *2 handfuls fresh/1 handful cooked, 1 medium piece* |
|  | Carrot  Apricot  Mango  Cantaloupe  Orange sweet potato | Hokkaido pumpkin  Butternut squash  Dark orange pumpkin | | Red bell pepper  Red grapefruit  Kaki  Chanterelle mushroom Milkcap mushroom | |  |  |  |  |  |  |  |  |  |  |
| **Q3** | **Other** vegetables, such as tomato, cucumber, onion, zucchini or eggplant, fresh, frozen, canned, cooked, fried (do not include potatoes, legumes or vegetables listed in previous questions) | | | | |  |  |  |  |  |  |  |  |  | *1 medium tomato, 1 handful cooked/2 handfuls fresh vegetables* |
| **Q4** | **Other** fruits, such as apples, berries, melons or oranges, whole or cut  (do NOT include fruit juices and smoothies) | | | | |  |  |  |  |  |  |  |  |  | *1 medium apple/orange, 1-2 slices melon, 1 handful berries* |
| **Q5** | White/yellow **potatoes** | | | | |  |  |  |  |  |  |  |  |  | *2-3 medium potatoes* |
| **Q6** | **White** bread, white bun or white roll | | | | |  |  |  |  |  |  |  |  |  | *2 slices of bread, 1 bun* |
| **Q7** | **White** rice, pasta/noodles, instant couscous, instant polenta or instant breakfast cereals (e.g. crisps, flakes, crunch) | | | | |  |  |  |  |  |  |  |  |  | *1 cup of cooked rice, pasta, couscous, polenta or instant breakfast cereals* |
| **Q8** | **Wholegrain** bread, bun or roll, **wholegrain** crackers or crispbread | | | | |  |  |  |  |  |  |  |  |  | *2 slices of*  *bread, 1 roll, 2-3 crackers/crispbreads* |
| **Q9** | Other **whole grains** (e.g. brown rice, brown pasta, grain kernels such as spelt, wheat, oats or barley, porridge, unsweetened wholegrain muesli, whole grain couscous, whole grain bulgur, quinoa, buckwheat or amaranth) | | | | |  |  |  |  |  |  |  |  |  | *1 cup cooked rice, pasta, porridge, kernels, couscous, bulgur, quinoa, buckwheat, amaranth, or muesli* |
| **Q10** | **Nuts and seeds**, such as walnuts, almonds, hazelnuts, pumpkin seeds, sunflower seeds or flaxseeds | | | | |  |  |  |  |  |  |  |  |  | *1 handful of nuts, 1 tablespoon of seeds* |
| **Q11** | **Nut and seed butters**, such as peanut butter or tahini | | | | |  |  |  |  |  |  |  |  |  | *1 tablespoon* |
|  |  | | | | | **Never** | **Rarely/**  **1x/month** | **2-3x/**  **month** | **1x/**  **week** | **2-3x/**  **week** | **4-6x/**  **week** | **1x/**  **day** | **2x/**  **day** | **≥3 times**  **/day** | **1 serving example** |
| **Q12** | **Vegan butter** or coconut oil | | | | |  |  |  |  |  |  |  |  |  | *1 tablespoon* |
| **Q13** | **Plant-based oils** such as olive, soybean, flaxseed or rapeseed oil (do NOT include here palm or coconut oil), **avocado** or **olives** | | | | |  |  |  |  |  |  |  |  |  | *1 tablespoon oil, 1/2 avocado, 5-10 olives* |
| **Q14** | **EPA/DHA (omega 3)-** fortified oils, EPA/DHA (omega 3), supplements or microalgae oil? | | | | |  |  |  |  |  |  |  |  |  | *1 tablespoon oil, 1 dose as per packaging instructions.* |
| **Q15** | **Traditional plant protein sources and derivates** like tofu, seitan, natto, tempeh, falafel, hummus, 100% red lentil or chickpea pasta, or soy cubes/granules | | | | |  |  |  |  |  |  |  |  |  | *½ small block tofu, seitan, tempeh, 4 falafel;1 cup cooked pasta, 1 small bowl  soy granules/ cubes , 2-3 tablespoons hummus* |
| **Q16** | **Beans, soybeans, lentils, chickpeas or peas** (excluding green peas and green beans) (do NOT include here their products, such as tofu, tempeh or hummus) | | | | |  |  |  |  |  |  |  |  |  | *1/2 cup cooked legumes* |
| **Q17** | **Packaged meat/fish alternatives** such as vegan salami, cold cuts, sausages, burger patties or fish fingers (excluding homemade recipes from raw sources)  **If 17=’never’, go to Q18** | | | | |  |  |  |  |  |  |  |  |  | *1 palm-sized piece, 1 sausage, 3-4 slices of salami etc.* |
| **Q17a** | When you buy these products, do you usually choose products low in salt? | | | | | Yes | No | Don’t know | | | | | | | |
| **Q18** | **Calcium-fortified plant-based milks, yogurts** (e.g., almond, soy, oat) or **calcium-set tofu** | | | | |  |  |  |  |  |  |  |  |  | *1 glass of milk, 1 cup yogurt, ½ small block of  tofu* |
| **Q19** | **Cheese alternatives**  **containing coconut oil**, such as sliced, solid or grated vegan cheese (e.g. vegan feta, mozzarella or cream cheese) | | | | |  |  |  |  |  |  |  |  |  | *1 slice of cheese, 1 tablespoon cream cheese or grated cheese* |
| **Q20** | **Savory snacks**, such as crisps/chips or salted crackers | | | | |  |  |  |  |  |  |  |  |  | *1 handful* |
| **Q21** | **Ready-to-eat meals** such as frozen pizza, croquettes, fried foods, spring rolls, dumplings, instant pasta or instant soup | | | | |  |  |  |  |  |  |  |  |  | *1 serving according to the package* |
| **Q22** | **Vegan sweets and desserts**, such as candy, “milk” chocolate, cake, ice cream or pudding | | | | |  |  |  |  |  |  |  |  |  | *1 piece of cake, 4 cookies, 1 handful of candy, 1 rip of chocolate, 1 scoop ice cream, 1 bowl of pudding* |
| **Q23** | **Sugar-sweetened beverages** such as soft/fizzy drinks, lemonades, sweetened ice tea, flavored plant-based milk, energy drinks, ginger beer or sports drinks | | | | |  |  |  |  |  |  |  |  |  | *1 glass* |
| **Q24** | **Artificially sweetened beverages**, such as diet/zero sugar soft/fizzy drinks, lemonades, energy drinks, “light” beverages, or sports drinks | | | | |  |  |  |  |  |  |  |  |  | *1 glass* |
| **Q25** | **Alcoholic beverages** such as beer, wine, cocktails or spirits | | | | |  |  |  |  |  |  |  |  |  | *1 can, 1 glass, 1 jigger/shot* |
|  |  | | | | | **Never** | **Rarely/**  **1x/month** | **2-3x/**  **month** | **1x/**  **week** | **2-3x/**  **week** | **4-6x/**  **week** | **1x/**  **day** | **2x/**  **day** | **≥3 times**  **/day** | **1 serving example** |

*These lists may be edited by adding country-specific fruits/vegetables, if they are: commonly consumed in a country AND if they contain >40mg Ca/100g AND have a low oxalate content (due to inhibited Ca absorption).

|  | **Do you regularly use:** | **Yes** | **No** |
| --- | --- | --- | --- |
| **Q26** | S**upplement for vitamin B12**  (either individually or as part of a multivitamin supplement) (e.g. pills, drops, injections, fortified toothpaste)? |  |  |
| **Q27** | I**odised salt** in food preparation, use an **iodine supplement**  (either individually or as part of a multimineral supplement) or consume **seaweed** to supplement for iodine intake (for Czech Republic also: iodine-rich mineral water) |  |  |
| **Q28** | **Vitamin D** **supplement**  (either individually or as part of a multivitamin supplement) during autumn and winter months? |  |  |
| **Q29** | **Selenium supplement** (either individually or as part of a multimineral supplement) or regularly consume **Brazil nuts**? |  |  |

**SOP 9 General Survey**

**SOCIODEMOGRAPHIC DATA**

SD1. How old are you? ____years

SD2. What is your gender identification?

1. Female
2. Male
3. Diverse

SD3. In which VEGANScreener study centre do you participate in?

1. Germany
2. Belgium
3. Spain
4. Czech Republic
5. Switzerland

SD4. Did you spend most of the last year in that country?

1. Yes
2. No

If no, what country did you spend most of the last year in?............

SD5. How would you tend to describe the area that you live in?

1. Rural
2. Urban

SD6. My highest diploma is:

1.Primary education or lower

2.Secundary education (including vocational education or vocational training)

3.Bacherlor or higher education

4.Other:_____________

SD7. With our total family income, our financial situation is:

1.  Very difficult

2.  Rather difficult

3.  Reasonable

            4.  Rather comfortable

5.  Very comfortable

SD8.  My professional situation is:

1. Paid work

2. Domestic work

3. Student

4. Voluntary work

5. Jobseeker

6. Other: ____________

SD9. What is your current marital status?

1. Single
2. Married or in partnership
3. Divorced
4. Widowed

SD10. How many live-in adults are there in your family? (18 years or older, including yourself)

___________

SD11. How many children are there in your family? (under 18 years) Fill below:

_________

**LIFESTYLE**

LS1. Do you or someone in your family prepare food cooking from scratch on a daily basis?

1. Yes
2. No

If yes, who prepares it more than 50 percent of the time?

1. Me myself
2. Someone else in my household

LS2. How many cups of coffee and black/green tea (combined) do you drink on average per week?

1. 0
2. 1-2
3. 3-4
4. 5-7
5. 8 or more

LS3. Do you currently smoke cigarettes?

1. NO, and I have never smoked
2. NO, but I am a former smoker
3. Yes, I currently smoke

LS4. How many alcoholic beverages do you drink per week? (expressed in equivalents equivalent to 1 small beer (330 ml), 1 small glass (1 dcL) of wine, 1 shot / jigger small of hard liquor, please give the total amount)

1. 0
2. 1-2
3. 3-6
4. 7 and more

LS5. How many hours a day do you sleep on average?

1. 0 - 4
2. 5
3. 6
4. 7
5. 8
6. more than 8

LS6. How often do you, on average, have trouble falling asleep, wake up too early, or have difficulties sleeping through the night?

1. Almost never
2. Once or twice in a few months
3. One to three times a month
4. Four or five times a month
5. Two or three times a week
6. More than 5 times a week - almost every night

**PHYSICAL ACTIVITY - IPAQ: physical activity in the LAST 7 DAYS.**

We are interested in finding out about the kinds of physical activities that people do as part of their everyday lives. The questions will ask you about the time you spent being physically active in the last 7 days. Please answer each question even if you do not consider yourself to be an active person. Please think about the activities you do at work, as part of your house and yard work, to get from place to place, and in your spare time for recreation, exercise or sport.

Think about all the vigorous activities that you did in the last 7 days. Vigorous physical activities refer to activities that take hard physical effort and make you breathe much harder than normal. Think only about those physical activities that you did for at least 10 minutes at a time

PA1. During the last 7 days, on how many days did you do vigorous physical activities like heavy lifting, digging, aerobics, or fast bicycling?

_____ days per week

**No high intensity activities (skip to question PA3)**

PA2. How much time does it take you to train on these days?

_____ hours per day

_____ minutes per day

Don’t know/Not sure

Think about all the moderate activities that you did in the last 7 days. Moderate activities refer to activities that take moderate physical effort and make you breathe somewhat harder than normal. Think only about those physical activities that you did for at least 10 minutes at a time.

PA3. How many days in the last 7 days have you spent doing moderate intensity activity such as gardening, cleaning, light exercise, rehabilitation exercises, ping pong, etc.? Do not include walking here.

_____ days per week

**No moderate activity (Skip to question PA5)**

PA4. How much time does this type of moderate activity take you on these days?

_____ hours per day

_____ minutes per day

 /Don't know/Not sure

Think about the time you spent walking in the last 7 days. This includes at work and at home, walking to travel from place to place, and any other walking that you have done solely for recreation, sport, exercise, or leisure.

PA5. How many days in the last 7 days did you spend walking that lasted at least 10 minutes at a time.

_____ days per week

**No walking (Skip to question PA7)**

PA6. How much time did you usually spend walking on one of those days?

_____ hours per day

_____ minutes per day

Don’t know/Not sure

The last question is about the time you spent sitting on weekdays during the last 7 days. Include time spent at work, at home, while doing course work and during leisure time. This may include time spent sitting at a desk, visiting friends, reading, or sitting or lying down to watch television.

PA7. During the last 7 days, how much time did you spend sitting on a week day?

_____ hours per day and  _____

minutes per day

Don’t know/Not sure

**DIET AND NUTRITION**

DN1. Over the past two years, with relation to your diet, you would describe yourself as:

1. Omnivore (your diet includes meat, fish, eggs, milk and/or other food of animal origin with no particular restrictions)
2. Flexitarian (you eat mainly foods of plant origin, but allow yourself to sometimes, on rare occasions, to eat some meat, fish, eggs or dairy)
3. Vegetarian (you do not eat meat and fish at all, but you consume milk and dairy products, eggs, …)
4. Vegan (you strictly follow a plant-based diet only, i.e. no consumption of meat, fish, dairy or eggs)

DN2. What table salt do you use in your household?

1. NO, we do not use any salt
2. Table salt (without added iodine)
3. Iodized table salt (with added iodine)
4. Iodized table salt with fluoride
5. Iodized table salt with folic acid
6. Iodized table salt with fluoride and folic acid
7. Others: ........................................................
8. Do not know or I have no preferences

*Note: Table salt refers to cooking salt, sea salt, rock salt, Himalayan salt, evaporated salt, etc.

DN3. I usually consume bakery products:

1. Bought
2. Home-made only

DN4. **(If answer to DN3 was “Home-made only)** If you ticked "Only home-made" in the previous question, then you use salt to make it:

1. Iodized

2. Without added iodine

DN5. Do you consume seaweed as a source of iodine?

1. NO, I do not consume seaweed

             2. YES

**(If answer to DN5 is “yes”)** How often do you consume seaweed?

2.1 Daily

2.2 Several times a week

2.3 Twice per week

2.4 Once per week

2.5 Monthly

2.6 Less frequently

DN6. Do you currently use dietary supplements (e.g., iron tablets, vitamin D drops, vitamin B12, iodine, DHA/EPA/fish-oil supplements, multivitamin/multimineral supplements)?

1. No
2. Yes

DN7. **If yes**, please name in each case the (manufacturer's) name of the supplement(s), dosage and how often you take this supplement. You have an example below.

(Fill in only the applicable boxes I.e. if you need less than the given boxes to enter your dietary supplement(s), the remaining boxes will be left blank).

| Supplement (name, brand) | All nutrient(s) included | Single dose/ amount | Frequency of consumption (average times month) |
| --- | --- | --- | --- |
| Watson Nutrition proveg omega 3+ | Vitamin D  Vitamin E  EPA  DHA  (EPA + DHA) | 1 capsule  25 µg/1000 IU  3 mg  Min. 75 mg  Min. 150 mg  (250 mg) | 30 |
|  |  |  |  |
|  |  |  |  |
|  |  |  |  |
|  |  |  |  |
|  |  |  |  |
|  |  |  |  |

DN8. Do you currently use fortified foods on a regular basis? (e.g., a calcium-fortified plant-based milk alternative, juice, oil). Do NOT include salt here.

1. Yes
2. No
3. Do not know

DN9. **If yes**, please name in each case what the (manufacturer's) name of the fortified product(s) is and how often you take this product.

(Fill in only the applicable boxes i.e. if you take less than 4 fortified products, leave the remaining boxes blank)

|  | Product name | Frequency of consumption (average per week) |
| --- | --- | --- |
| 1. Fortified product |  |  |
| 2. Fortified product |  |  |
| 3. Fortified product |  |  |
| 4. Fortified product |  |  |

**QUESTIONS FOR PEOPLE EATING WITHOUT RESTRICTION OF ANIMAL PRODUCTS (DN10-DN13)**

DN10. How often do you consume meat and/or meat products (e.g., sausage)?*

***This does not include plant-based meat alternatives**

1. Never
2. Less than 1 time per month
3. 1-3 times per month
4. 1 time per week
5. 2 times per week
6. 3-4 times per week
7. 5-6 times per week
8. Daily
9. Several times a day

DN11. How often do you consume fish/fish products? *

***This does not include plant-based fish alternatives**

1. Never
2. Less than 1 time per month
3. 1-3 times per month
4. 1 time per week
5. 2 times per week
6. 3-4 times per week
7. 5-6 times per week
8. Daily
9. Several times a day

DN12. How often do you consume milk/dairy products?

***This does not include plant-based milk alternatives.**

1. Never
2. Less than 1 time per month
3. 1-3 times per month
4. 1 time per week
5. 2 times per week
6. 3-4 times per week
7. 5-6 times per week
8. Daily
9. Several times a day

DN13. How often do you consume egg/egg products (meaning also eggs contained in e.g. cakes, pasta or other ready-made products)?

***This does not include plant-based egg alternatives**

1. Never
2. Less than 1 time per month
3. 1-3 times per month
4. 1 time per week
5. 2 times per week
6. 3-4 times per week
7. 5-6 times per week
8. Daily
9. Several times a day

**QUESTIONS FOR VEGANS ONLY (DN14-DN18)**

DN14. Counting back from today, how many consecutive years are you adhering to your vegan diet? (Do not include any periods of vegetarian or flexitarian or reductarian diet)

...................................................... (years)

DN15. When did you start your current vegan diet? Date: _____

DN16. Are there occasions when you exceptionally consume meat, fish, milk, dairy products, eggs, and/or honey?

1. Yes
2. No

DN17. Why do you eat vegan? Please select the motive that is most important to you.

1. For ethical/animal rights motives (rejection of factory farming/animal use, right to life for animals, emotional attachment to animals, etc.).
2. For health reasons
3. For ecological reasons (climate protection, preservation of resources, etc.)
4. For reasons of world nutrition/social justice
5. For religious reasons
6. For other reasons (e.g. dislike or disgust of meat/animal products)
7. Other, please list: ______________________________________________________

DN18. Which other motives apply to you?

**Please select all that apply:**

1. Ethical/animal rights motives (rejection of factory farming/animal use, right to life for animals, emotional attachment to animals, etc.).
2. Health reasons
3. Ecological reasons (climate protection, preservation of resources)
4. World nutrition/social justice
5. Religious reasons
6. Other reasons (e.g. disgust of meat/animal products)
7. None of the motives mentioned
8. Other, please list:______________________________________________________

**SOP 10 Well-being Questionnaires**

**SECURE FLOURISH MEASURE**

**Please respond to the following questions on a scale from 0 to 10:**

1. Overall, how satisfied are you with life as a whole these days?

0 = Not Satisfied at All, 10 = Completely Satisfied

2. In general, how happy or unhappy do you usually feel?

0 = Extremely Unhappy, 10 = Extremely Happy

3. In general, how would you rate your physical health?

0 = Poor, 10 = Excellent

4. How would you rate your overall mental health?

0 = Poor, 10 = Excellent

5. Overall, to what extent do you feel the things you do in your life are worthwhile?

0 = Not at All Worthwhile, 10 = Completely Worthwhile

6. I understand my purpose in life.

0 = Strongly Disagree, 10 = Strongly Agree

7. I always act to promote good in all circumstances, even in difficult and challenging

situations.

0 = Not True of Me, 10 = Completely True of Me

8. I am always able to give up some happiness now for greater happiness later.

0 = Not True of Me, 10 = Completely True of Me

9. I am content with my friendships and relationships.

0 = Strongly Disagree, 10 = Strongly Agree

10. My relationships are as satisfying as I would want them to be.

0 = Strongly Disagree, 10 = Strongly Agree

11. How often do you worry about being able to meet normal monthly living expenses?

0 = Worry All of the Time, 10 = Do Not Ever Worry

12. How often do you worry about safety, food, or housing?

0 = Worry All of the Time, 10 = Do Not Ever Worry

**BECK DEPRESSION INVENTORY II (BDI-II)**

**Instructions:** This questionnaire consists of 21 groups of statements. Please read each group of states carefully, and state and then pick out the one statement in each group that best describes the way you have been feeling during the past two weeks, including today. Select the statement you have picked if several statements in the group seem to apply equally well, select the statement with the highest number for that group.

**1.-Sadness**

0 I do not feel sad.

1 I feel sad much of the time.

2 I am sad all the time.

3 I am so sad or unhappy that I can´t stand it.

**2.-Pessimism**

0 I am not discouraged about my future.

1 I feel more discouraged about my future than I used to be.

2 I do not expect things to work out for me.

3 I feel my future is hopeless and will only get worse.

**3.-Past Failure**

0 I do not feel like a failure.

1 I have failed more than I should have.

2 As I look back, I see a lot of failures.

3 I feel I am a total failure as a person.

**4.-Loss of Pleasure**

0 I get as much pleasure as I ever did from the things I used to enjoy.

1 I don’t enjoy things as much as I used to.

2 I get very little pleasure from the things I used to enjoy.

3 I can’t get any pleasure from the thing I used to enjoy.

**5.-Guilty Feeling**

0 I don´t feel particularly guilty.

1 I feel guilty over many things I have done or should have done.

2 I feel quite guilty most of the time.

3 I feel guilty all of the time.

**6.-Punishment Feelings**

0 I don´t feel I am being punished.

1 I feel I may be punished.

2 I expect to be punished.

3 I feel I am being punished.

**7.-Self-Dislike**

0 I feel the same about myself as ever.

1 I have lost confidence in myself.

2 I am disappointed in myself.

3 I dislike myself.

**8.-Self-Criticalness**

0 I don´t criticize or blame myself more than usual.

1 I am more critical of myself than I used to be.

2 I criticize myself for all of my faults.

3 I blame myself for everything bad that happens

**9. Suicidal thoughts or Whishes**

0 I don´t have any thoughts of killing myself.

1 I have thoughts of killing myself, but I would not carry them out.

2 I would like to kill myself.

3 I would kill myself if I had the chance.

**10. Crying**

0 I don´t cry any more than I used to.

1 I cry more than I used to.

2 I cry over every little thing.

3 I feel like crying, but I can’t.

**11. Agitation**

0 I am no more restless or wound up than usual.

1 I feel more restless or wound up than usual.

2 I am so restless or agitated that it’s hard to stay still.

3 I am so restless or agitated than I have to keep moving or doing something.

**12. Loss of interests**

0 I have not lost interest in other people or activities.

1 I am less interested in other people or things than before.

2 I have lost most of my interest in other people or things.

3 It's hard to get interested in anything.

**13. Indecisiveness**

0 I make decisions about as well as ever.

1 I find it more difficult to make decisions than usual.

2 I have much greater difficulty in making decisions than I used to.

3 I have trouble making any decisions.

**14. Worthlessness**

0 I do not feel I am worthless.

1 I don't consider myself as worthwhile and useful as I used to.

2 I feel more worthless as compared to other people.

3 I feel utterly worthless.

**15. Loss of Energy**

0 I have as much energy as ever.

1 I have less energy than I used to have.

2 I don't have enough energy to do very much.

3 I don't have enough energy to do anything.

**16. Changes in Sleeping Pattern**

0 I have not experienced any change in my sleeping pattern.

1 I sleep somewhat more than usual. / I sleep somewhat less than usual.

2 I sleep a lot more than usual. / I sleep a lot less than usual.

3 I sleep most of the day. / I wake up 1-2 hours early and can't get back to sleep.

**17. Irritability**

0 I am no more irritable than usual

1 I am more irritable than usual

2 I am much more irritable than usual

3 I am irritable all the time

**18. Changes in Appetite**

0 I have not experienced any change in my appetite

1 My appetite is somewhat less than usual. / My appetite is somewhat more than usual.

2 My appetite is much less than before. / My appetite is much greater than usual.

3 I have no appetite at all. / I crave food all the time.

**19. Concentration Difficulty**

0 I can concentrate as well as ever.

1 I can't concentrate as well as usual.

2 It's hard to keep my mind on anything for very long.

3 I find I can't concentrate on anything.

**20. Tiredness or Fatigue**

0 I am no more tired or fatigued than usual.

1 I get more tired or fatigued more easily than usual.

2 I am too tired or fatigued to do a lot of the things I used to do.

3 I am too tired or fatigued to do most of the things I used to do.

**21. Loss of interest in Sex**

0 I have not noticed any recent change in my interest in sex.

1 I am less interested in sex than I used to be.

2 I am much less interested in sex now.

3 I have lost interest in sex completely.

**EXTRA QUESTION**

**In general, would you say your health is:**

□1 Excellent □2 Very good □3 Good □4 Fair □5 Poor

**SOP 11 Food Frequency Questionnaire**

Indicate your average consumption over the past year for each of the following **foods** and **beverages** as best as you can by ticking (✓) EVERY LINE – do not leave out ANY items. Please also take into account seasonal variations. **For example**, if you eat ice cream 4 times/week only during the summer (three months), the average consumption per year is 1 time/week.

**FOR BEVERAGES:** Select the frequency with which you have consumed each drink, on average over the past year. Use the serving sizes below for all drinks except you do not use the serving size listed. **For example**, if you consume **1 can of coca-cola 4 times a week**, as **1 can (330mL)** equals to **1.5 servings**, the answer to indicate would be **5-6 times per week (4x1.5=6).**

**NOTE:** For more details, please see the food frequency questionnaire instructions.

|  |  | **AVERAGE USE LAST YEAR** | | | | | | | | |
| --- | --- | --- | --- | --- | --- | --- | --- | --- | --- | --- |
|  | **FOODS AND AMOUNTS** | **Never or less than once a month** | **1-3 per month** | **Once a week** | **2-4 per week** | **5-6 per week** | **Once a day** | **2-3 per day** | **4-5 per day** | **6+ per day** |
|  | **MILK /DAIRY PRODUCTS** |  |  |  |  |  |  |  |  |  |
| 1 | Full cream/whole milk  (1 cup, 200mL) |  |  |  |  |  |  |  |  |  |
| 2 | Semi-skimmed milk  (1 cup, 200mL) |  |  |  |  |  |  |  |  |  |
| 3 | Skimmed milk  (1 cup, 200mL) |  |  |  |  |  |  |  |  |  |
| 4 | Milk shake (1 cup, 200mL) |  |  |  |  |  |  |  |  |  |
| 5 | Condensed milk (1 tablespoon, 20g) |  |  |  |  |  |  |  |  |  |
| 6 | Single or sour cream (1 tablespoon, 20g) |  |  |  |  |  |  |  |  |  |
| 7 | Double or clotted cream (1 tablespoon, 20g) |  |  |  |  |  |  |  |  |  |
| 8 | Yogurt Full fat or Greek yogurt (1 carton, 125g) |  |  |  |  |  |  |  |  |  |
| 9 | Low fat yogurt (1 carton, 125g) |  |  |  |  |  |  |  |  |  |
| 10 | Dairy desserts (custard, flan, pudding) (1 carton, 125g) |  |  |  |  |  |  |  |  |  |
| 11 | Cottage cheese, curds (1/2 cup), low fat soft cheese (40g) |  |  |  |  |  |  |  |  |  |
| 12 | Cream cheese (25g) |  |  |  |  |  |  |  |  |  |
| 13 | Cheese, e.g., Cheddar, Brie, Edam (40g) |  |  |  |  |  |  |  |  |  |
| 14 | Fresh, white cheese (50g) |  |  |  |  |  |  |  |  |  |
|  | **FOODS AND AMOUNTS** | **Never or less than once a month** | **1-3 per month** | **Once a week** | **2-4 per week** | **5-6 per week** | **Once a day** | **2-3 per day** | **4-5 per day** | **6+ per day** |
|  | **PLANT-BASED MILK AND DAIRY SUBSTITUTES** |  |  |  |  |  |  |  |  |  |
| 15 | Almond, hazelnut milk (1 cup, 200mL) |  |  |  |  |  |  |  |  |  |
| 16 | Calcium-enriched almond, hazelnut milk (1 cup, 200mL) |  |  |  |  |  |  |  |  |  |
| 17 | Soy milk  (1 cup, 200mL) |  |  |  |  |  |  |  |  |  |
| 18 | Calcium-enriched soy enriched milk  (1 cup, 200mL) |  |  |  |  |  |  |  |  |  |
| 19 | Rice, Oat milk  (1 cup, 200mL) |  |  |  |  |  |  |  |  |  |
| 20 | Calcium-enriched rice, oat milk  (1 cup, 200mL) |  |  |  |  |  |  |  |  |  |
| 21 | Vegan cooking cream (1 tablespoon, 20g) |  |  |  |  |  |  |  |  |  |
| 22 | Soy yogurt  (1 carton, 125g) |  |  |  |  |  |  |  |  |  |
| 23 | Calcium-enriched soy yogurt  (1 carton, 125g) |  |  |  |  |  |  |  |  |  |
| 24 | Calcium-enriched almond or coconut yogurt (1 carton, 125g) |  |  |  |  |  |  |  |  |  |
| 25 | Plant-based desserts (custard, pudding) (1 carton, 125g) |  |  |  |  |  |  |  |  |  |
| 26 | Vegan cream cheese (25g), blue cheese, vegan mozzarella, vegan cheddar (40g) |  |  |  |  |  |  |  |  |  |

|  | **FOODS AND AMOUNTS** | **AVERAGE USE LAST YEAR** | | | | | | | | |
| --- | --- | --- | --- | --- | --- | --- | --- | --- | --- | --- |
|  | **EGGS, MEAT AND FISH**  A plate or serving (100-150 g) except where other quantities are indicated | **Never or less than once a month** | **1-3 per month** | **Once a week** | **2-4 per week** | **5-6 per week** | **Once a day** | **2-3 per day** | **4-5 per day** | **6+ per day** |
| 27 | Eggs (1 unit, 50g) |  |  |  |  |  |  |  |  |  |
| 28 | Beef (1 serving) |  |  |  |  |  |  |  |  |  |
| 29 | Burger (1 serving, 60g) |  |  |  |  |  |  |  |  |  |
| 30 | Pork (1 serving) |  |  |  |  |  |  |  |  |  |
| 31 | Lamb (1 serving) |  |  |  |  |  |  |  |  |  |
| 32 | Chicken or turkey (with skin) (1 serving) |  |  |  |  |  |  |  |  |  |
| 33 | Chicken or turkey (without skin) (1 serving) |  |  |  |  |  |  |  |  |  |
| 34 | Rabbit (1 serving) |  |  |  |  |  |  |  |  |  |
| 35 | Liver (beef, pork, chicken) (1 serving) |  |  |  |  |  |  |  |  |  |
| 36 | Other viscera (brain, heart, gizzard) (1 serving) |  |  |  |  |  |  |  |  |  |
| 37 | Liver paté, foie-gras (25g) |  |  |  |  |  |  |  |  |  |
| 38 | Bacon, streaky bacon (50g) |  |  |  |  |  |  |  |  |  |
| 39 | Ham, cooked ham (1 slice, 30g) |  |  |  |  |  |  |  |  |  |
| 40 | Turkey ham (1 slice, 30g) |  |  |  |  |  |  |  |  |  |
| 41 | Serrano ham (1 slice, 30g) |  |  |  |  |  |  |  |  |  |
| 42 | Processed meat e.g., sausages, chorizo, mortadella, blood pudding, (For Germany and CZ: sausages, chorizo, mortadella, blood pudding, spreadable meat) (40g) |  |  |  |  |  |  |  |  |  |
| 43 | White fish, fresh or frozen, e.g., haddock, plaice, sole, halibut (1 serving) (Spain: hake, whiting, sea bream, grouper, sole) |  |  |  |  |  |  |  |  |  |
| 44 | Oily fish, fresh or frozen, e.g., salmon, tuna, mackerel, sardine) (1 serving) (Spain: salmon, bonito, tuna, mackerel, sardines) |  |  |  |  |  |  |  |  |  |
| 45 | Oysters, clams and mussels (6 units) |  |  |  |  |  |  |  |  |  |
| 46 | Squid, octopus, cuttlefish (1 serving, 200g) |  |  |  |  |  |  |  |  |  |
| 47 | Prawns, langoustines (4-5 units, 200g) |  |  |  |  |  |  |  |  |  |
| 48 | Canned fish and seafood in oil (sardines, anchovies, bonito, tuna) (1 small can, 50g) |  |  |  |  |  |  |  |  |  |
| 49 | Canned fish and seafood natural (sardines, anchovies, bonito, tuna) (1 small can, 50g) |  |  |  |  |  |  |  |  |  |
|  |  | **AVERAGE USE LAST YEAR** | | | | | | | | |
|  | **FOODS AND AMOUNTS** | **Never or less than once a month** | **1-3 per month** | **Once a week** | **2-4 per week** | **5-6 per week** | **Once a day** | **2-3 per day** | **4-5 per day** | **6+ per day** |
|  | **EGG, MEAT AND FISH SUBSTITUTES** |  |  |  |  |  |  |  |  |  |
| 50 | Egg Replacer (1 tablespoon, 20g) |  |  |  |  |  |  |  |  |  |
| 51 | Seitan (½ small block, 100g) |  |  |  |  |  |  |  |  |  |
| 52 | Texturized vegetable protein (from dry) (3 tablespoons, 30g) |  |  |  |  |  |  |  |  |  |
| 53 | Tofu-silken/soft (60g) |  |  |  |  |  |  |  |  |  |
| 54 | Tofu-firm (60g) |  |  |  |  |  |  |  |  |  |
| 55 | Tempeh (½ small block, 100g) |  |  |  |  |  |  |  |  |  |
| 56 | Natto (60g) |  |  |  |  |  |  |  |  |  |
| 57 | Vegan pate (25g) |  |  |  |  |  |  |  |  |  |
| 58 | Vegan processed meat e.g., sausages, chorizo, and mortadella (For Germany and CZ: sausages, chorizo, and mortadella, spreadable meat alternative) (40g) |  |  |  |  |  |  |  |  |  |
| 59 | Vegan burger (one serving, 60g) |  |  |  |  |  |  |  |  |  |
| 60 | Prepacked vegan fish e.g., fish sticks, fish filets, crab or fish cakes, vegan tuna (120g) |  |  |  |  |  |  |  |  |  |
| 61 | Prepacked vegan salmon, vegan squid, prawns (1 serving 200g) |  |  |  |  |  |  |  |  |  |

Other plant-based foods consumed frequently? List them below, only one food per line for one to five most frequently consumed and the portion eaten.

| **Food** | **Portion eaten (one serving)** | **Average use last year** |
| --- | --- | --- |
|  |  | 1, Never or less than once a month  2, 1-3 per month  3, Once a week  4, 2-4 per week  5, 5-6 per week  6, Once a day  7, 2-3 per day  8, 4-5 per day  9, 6+ per day |
|  |  | 1, Never or less than once a month  2, 1-3 per month  3, Once a week  4, 2-4 per week  5, 5-6 per week  6, Once a day  7, 2-3 per day  8, 4-5 per day  9, 6+ per day |
|  |  | 1, Never or less than once a month  2, 1-3 per month  3, Once a week  4, 2-4 per week  5, 5-6 per week  6, Once a day  7, 2-3 per day  8, 4-5 per day  9, 6+ per day |
|  |  | 1, Never or less than once a month  2, 1-3 per month  3, Once a week  4, 2-4 per week  5, 5-6 per week  6, Once a day  7, 2-3 per day  8, 4-5 per day  9, 6+ per day |
|  |  | 1, Never or less than once a month  2, 1-3 per month  3, Once a week  4, 2-4 per week  5, 5-6 per week  6, Once a day  7, 2-3 per day  8, 4-5 per day  9, 6+ per day |

|  |  | **AVERAGE USE LAST YEAR** | | | | | | | | |
| --- | --- | --- | --- | --- | --- | --- | --- | --- | --- | --- |
|  | **FOODS AND AMOUNTS** | **Never or less than once a month** | **1-3 per month** | **Once a week** | **2-4 per week** | **5-6 per week** | **Once a day** | **2-3 per day** | **4-5 per day** | **6+ per day** |
|  | **BREAD AND SAVOURY BISCUITS** (one slice or biscuit) |  |  |  |  |  |  |  |  |  |
| 62 | White bread and rolls |  |  |  |  |  |  |  |  |  |
| 63 | Wholemeal bread and rolls |  |  |  |  |  |  |  |  |  |
| 64 | Cream crackers, cheese biscuits |  |  |  |  |  |  |  |  |  |
| 65 | Crispbread |  |  |  |  |  |  |  |  |  |
| 66 | **CEREALS AND PASTA** |  |  |  |  |  |  |  |  |  |
| 67 | Porridge, readybrek (1 bowl, 200g) |  |  |  |  |  |  |  |  |  |
| 68 | Breakfast cereal (crisps, flakes, crunch) (30g) |  |  |  |  |  |  |  |  |  |
| 69 | Breakfast wholemeal cereal (muesli, granola) (30g) |  |  |  |  |  |  |  |  |  |
| 70 | Quinoa (60g in raw) |  |  |  |  |  |  |  |  |  |
| 71 | Buckwheat, amaranth (60g in raw) |  |  |  |  |  |  |  |  |  |
| 72 | Spelt (60g in raw) |  |  |  |  |  |  |  |  |  |
| 73 | Barley (60g in raw) |  |  |  |  |  |  |  |  |  |
| 74 | Bulgur (60g in raw) |  |  |  |  |  |  |  |  |  |
| 75 | Millet (60g in raw) |  |  |  |  |  |  |  |  |  |
| 76 | Couscous, instant polenta (60g in raw) |  |  |  |  |  |  |  |  |  |
| 77 | White rice (60g in raw) |  |  |  |  |  |  |  |  |  |
| 78 | Brown rice (60g in raw) |  |  |  |  |  |  |  |  |  |
| 79 | White or green pasta e.g., spaghetti, macaroni, noodles (60g in raw) |  |  |  |  |  |  |  |  |  |
| 80 | Wholemeal pasta (60g in raw) |  |  |  |  |  |  |  |  |  |
| 81 | Pizza (1 serving, 200g) |  |  |  |  |  |  |  |  |  |
| 82 | Vegan pizza (1 serving, 200g) |  |  |  |  |  |  |  |  |  |

|  |  | **AVERAGE USE LAST YEAR** | | | | | | | | |
| --- | --- | --- | --- | --- | --- | --- | --- | --- | --- | --- |
|  | **FOODS AND AMOUNTS** | **Never or less than once a month** | **1-3 per month** | **Once a week** | **2-4 per week** | **5-6 per week** | **Once a day** | **2-3 per day** | **4-5 per day** | **6+ per day** |
|  | **SWEETS, PASTRIES AND SNACKS** |  |  |  |  |  |  |  |  |  |
| 83 | Sweet biscuits, chocolate (4 units, 50g) |  |  |  |  |  |  |  |  |  |
| 84 | Sweet biscuits, plain (4-6 units, 50g) |  |  |  |  |  |  |  |  |  |
| 85 | Whole-grain or fibre biscuits (4-6 units, 50g) |  |  |  |  |  |  |  |  |  |
| 86 | Rice or corn cakes (2 units) |  |  |  |  |  |  |  |  |  |
| 87 | Homemade cakes (50g) |  |  |  |  |  |  |  |  |  |
| 88 | ﻿Non-homemade pastries and confectionery e.g., cake (1 slice), muffin, doughnut, croissant (1 unit) |  |  |  |  |  |  |  |  |  |
| 89 | Vegan homemade cakes (50g) |  |  |  |  |  |  |  |  |  |
| 90 | Non-homemade vegan pastries and confectionery e.g., cake (1 slice), muffin, doughnut (1 unit) |  |  |  |  |  |  |  |  |  |
| 91 | Ice cream (1 unit, 60g) |  |  |  |  |  |  |  |  |  |
| 92 | Vegan ice cream (1 unit, 60g) |  |  |  |  |  |  |  |  |  |
| 93 | Chocolate, single or squares (1 ounce) |  |  |  |  |  |  |  |  |  |
| 94 | Chocolate snack bars (1 unit) |  |  |  |  |  |  |  |  |  |
| 95 | Cocoa powder (1 teaspoon) |  |  |  |  |  |  |  |  |  |
| 96 | Sweets, mints (1 unit) |  |  |  |  |  |  |  |  |  |
| 97 | Crisps or other packet snacks. e.g., popcorn, chips (tortilla-corn), pretzel, including vegan options  (1 packet, 50g) |  |  |  |  |  |  |  |  |  |
| 98 | Others (1 serving):  1. |  |  |  |  |  |  |  |  |  |
|  | 2. |  |  |  |  |  |  |  |  |  |
|  |  | **AVERAGE USE LAST YEAR** | | | | | | | | |
|  | **FOODS AND AMOUNTS** | **Never or less than once a month** | **1-3 per month** | **Once a week** | **2-4 per week** | **5-6 per week** | **Once a day** | **2-3 per day** | **4-5 per day** | **6+ per day** |
|  | **SOUPS, SAUCES, SPREADS, SUGAR, SALT, OTHERS** |  |  |  |  |  |  |  |  |  |
| 99 | Ready to eat vegetable soups (1 unit) |  |  |  |  |  |  |  |  |  |
| 100 | Ready to eat meat soups (1 unit) |  |  |  |  |  |  |  |  |  |
| 101 | Sauces, e.g., white sauce, cheese sauce, gravy (60g) |  |  |  |  |  |  |  |  |  |
| 102 | Tomato ketchup (1 tablespoon, 15g) |  |  |  |  |  |  |  |  |  |
| 103 | Homemade sofrito (sauce of tomato, garlic, onions or leeks sautéed in olive  oil) (1 tablespoon) |  |  |  |  |  |  |  |  |  |
| 104 | Mayonnaise (1 tablespoon, 30g) |  |  |  |  |  |  |  |  |  |
| 105 | Vegan mayonnaise (1 tablespoon, 30g) |  |  |  |  |  |  |  |  |  |
| 106 | Hummus (2-3 tablespoons) |  |  |  |  |  |  |  |  |  |
| 107 | Jam, marmalade (1 tablespoon, 15g) |  |  |  |  |  |  |  |  |  |
| 108 | Honey (1 tablespoon, 20g) |  |  |  |  |  |  |  |  |  |
| 109 | Sugar (1 teaspoon, 5g) |  |  |  |  |  |  |  |  |  |
| 110 | Sweeteners (1 serving) |  |  |  |  |  |  |  |  |  |
| 111 | Salt (1 pinch) |  |  |  |  |  |  |  |  |  |
| 112 | Croquettes, pasties (1 unit) |  |  |  |  |  |  |  |  |  |
| 113 | Vegan croquettes, pasties (1 unit) |  |  |  |  |  |  |  |  |  |

|  |  | **AVERAGE USE LAST YEAR** | | | | | | | | |
| --- | --- | --- | --- | --- | --- | --- | --- | --- | --- | --- |
|  | **FOODS AND AMOUNTS** | **Never or less than once a month** | **1-3 per month** | **Once a week** | **2-4 per week** | **5-6 per week** | **Once a day** | **2-3 per day** | **4-5 per day** | **6+ per day** |
|  | **DRINKS** |  |  |  |  |  |  |  |  |  |
| 113 | Tap water (1 glass, 250mL) |  |  |  |  |  |  |  |  |  |
| 114 | Calcium rich mineral water (≥ 300mg/L) (1 glass, 250mL) |  |  |  |  |  |  |  |  |  |
| 115 | Other bottled water (1 glass, 250 ml) |  |  |  |  |  |  |  |  |  |
| 116 | Tea (1 cup, 150mL) |  |  |  |  |  |  |  |  |  |
| 117 | Coffee, instant or ground (1 cup, 50mL) |  |  |  |  |  |  |  |  |  |
| 118 | Coffee, decaffeinated instant or ground (1 cup, 50mL) |  |  |  |  |  |  |  |  |  |
| 119 | Coffee whitener, e.g., Coffee-mate (1 teaspoon) |  |  |  |  |  |  |  |  |  |
| 120 | Hot chocolate (1 cup, 150mL) |  |  |  |  |  |  |  |  |  |
| 121 | Red wine (1 glass, 125mL) |  |  |  |  |  |  |  |  |  |
| 122 | White or Rose wine (1 glass, 125mL) |  |  |  |  |  |  |  |  |  |
| 123 | Beer, lager or cider  (1 glass, 200mL) |  |  |  |  |  |  |  |  |  |
| 124 | Port, sherry, vermouth, liqueurs, sweet wine (1 glass, 50mL) |  |  |  |  |  |  |  |  |  |
| 125 | Spirits, e.g., gin, brandy, whisky, vodka (single, 20mL) |  |  |  |  |  |  |  |  |  |
| 126 | Low calorie or diet fizzy soft drinks (1 glass, 200mL) |  |  |  |  |  |  |  |  |  |
| 127 | Fizzy soft drinks, e.g., Coca-cola, lemonade or others (1 glass, 200mL) |  |  |  |  |  |  |  |  |  |
| 128 | Fresh orange juice (homemade) (1 glass, 200mL) |  |  |  |  |  |  |  |  |  |
| 129 | Other fresh juices (homemade) (1 glass, 200mL) |  |  |  |  |  |  |  |  |  |
| 130 | bottled or canned fruit or vegetable juices (1 glass, 200mL) |  |  |  |  |  |  |  |  |  |
| 131 | Other drinks (1 glass, 200mL): |  |  |  |  |  |  |  |  |  |
|  | 1. |  |  |  |  |  |  |  |  |  |
|  | 2. |  |  |  |  |  |  |  |  |  |

|  |  | **AVERAGE USE LAST YEAR** | | | | | | | | |
| --- | --- | --- | --- | --- | --- | --- | --- | --- | --- | --- |
|  | **FOODS AND AMOUNTS** | **Never or less than once a month** | **1-3 per month** | **Once a week** | **2-4 per week** | **5-6 per week** | **Once a day** | **2-3 per day** | **4-5 per day** | **6+ per day** |
|  | **FRUIT AND SEED**  Estimate your average use when the fruit is in season |  |  |  |  |  |  |  |  |  |
| 132 | Apples or pears (1 medium size, 200g) |  |  |  |  |  |  |  |  |  |
| 133 | Oranges (1 unit), satsumas or mandarins (2 units) |  |  |  |  |  |  |  |  |  |
| 134 | Grapefruit (half, 80g) |  |  |  |  |  |  |  |  |  |
| 135 | Bananas (1 unit, 100g) |  |  |  |  |  |  |  |  |  |
| 136 | Grapes (medium serving, 100g) |  |  |  |  |  |  |  |  |  |
| 137 | Pineapple (1 slice, 120g) |  |  |  |  |  |  |  |  |  |
| 138 | Melon, cantaloupe (1 slice, 175g) |  |  |  |  |  |  |  |  |  |
| 139 | Peach (1 small piece), apricot (3 pieces), nectarine (1 small piece) |  |  |  |  |  |  |  |  |  |
| 140 | Strawberries, raspberries, blueberries (medium serving, 80g) |  |  |  |  |  |  |  |  |  |
| 141 | Cherries (medium serving, 80g), plums (2 units, 120g) |  |  |  |  |  |  |  |  |  |
| 142 | Kiwi fruit (medium serving, 60g) |  |  |  |  |  |  |  |  |  |
| 143 | Mango (120g), papaya (200g), khaki (1 unit) |  |  |  |  |  |  |  |  |  |
| 144 | Tinned fruit in juice or syrup (2 units) |  |  |  |  |  |  |  |  |  |
| 145 | Dried fruit, e.g., dates (3 units), dried fig (2 units), raisins, prunes (medium serving, 30g) |  |  |  |  |  |  |  |  |  |
| 146 | Walnuts (1 handful, 30g) |  |  |  |  |  |  |  |  |  |
| 147 | Cashew nuts (1 handful, 30g) |  |  |  |  |  |  |  |  |  |
| 148 | Almonds (1 handful, 30g) |  |  |  |  |  |  |  |  |  |
| 149 | hazelnuts (1 handful, 30g) |  |  |  |  |  |  |  |  |  |
| 150 | Peanuts (1 handful, 30g) |  |  |  |  |  |  |  |  |  |
| 151 | Pistachio (1 handful, 30g) |  |  |  |  |  |  |  |  |  |
| 152 | Pecans (1 handful, 30g) |  |  |  |  |  |  |  |  |  |
| 153 | Brazil nuts (1 handful, 30g) |  |  |  |  |  |  |  |  |  |
| 154 | Dried coconut (45g) |  |  |  |  |  |  |  |  |  |
| 155 | Pumpkin seeds (2 tablespoons, 20g) |  |  |  |  |  |  |  |  |  |
| 156 | Sunflower seeds (2 tablespoons, 20g) |  |  |  |  |  |  |  |  |  |
| 157 | Sesame seeds (2 tablespoons, 20g) |  |  |  |  |  |  |  |  |  |
| 158 | Flax seeds (1 tablespoon, 10g) |  |  |  |  |  |  |  |  |  |
| 159 | Olives (10 units) |  |  |  |  |  |  |  |  |  |
| 160 | Avocado (1/2 of a medium size) |  |  |  |  |  |  |  |  |  |
| 161 | Other fruit (1 unit) or seed (2 tablespoons, 20g): |  |  |  |  |  |  |  |  |  |
|  | 1. |  |  |  |  |  |  |  |  |  |
|  | 2. |  |  |  |  |  |  |  |  |  |
|  |  | **AVERAGE USE LAST YEAR** | | | | | | | | |
|  | **FOODS AND AMOUNTS** | **Never or less than once a month** | **1-3 per month** | **Once a week** | **2-4 per week** | **5-6 per week** | **Once a day** | **2-3 per day** | **4-5 per day** | **6+ per day** |
|  | **Seeds/Nut Butters** |  |  |  |  |  |  |  |  |  |
| 162 | Cashew butter (20g) |  |  |  |  |  |  |  |  |  |
| 163 | Almond butter (20g) |  |  |  |  |  |  |  |  |  |
| 164 | Peanut butter (20g) |  |  |  |  |  |  |  |  |  |
| 165 | Tahini (sesame butter) (20g) |  |  |  |  |  |  |  |  |  |

|  |  | **AVERAGE USE LAST YEAR** | | | | | | | | |
| --- | --- | --- | --- | --- | --- | --- | --- | --- | --- | --- |
|  | **FOODS AND AMOUNTS** | **Never or less than once a month** | **1-3 per month** | **Once a week** | **2-4 per week** | **5-6 per week** | **Once a day** | **2-3 per day** | **4-5 per day** | **6+ per day** |
|  | **VEGETABLES** (Fresh, frozen or tinned) |  |  |  |  |  |  |  |  |  |
| 166 | Carrots (1 medium size, 80g) |  |  |  |  |  |  |  |  |  |
| 167 | Spinach, chard (1 plate, 200 g) |  |  |  |  |  |  |  |  |  |
| 168 | Broccoli, bok choy, spring greens, kale (1 plate, 150 g) |  |  |  |  |  |  |  |  |  |
| 169 | Brussels sprouts (6 units) |  |  |  |  |  |  |  |  |  |
| 170 | Cabbage (1 plate, 150 g) |  |  |  |  |  |  |  |  |  |
| 171 | Zucchini, aubergine (90g) |  |  |  |  |  |  |  |  |  |
| 172 | Hokkaido pumpkin, butternut squash (1 plate, 200 g) |  |  |  |  |  |  |  |  |  |
| 173 | Cauliflower (1 plate, 150 g) |  |  |  |  |  |  |  |  |  |
| 174 | Green peas (1 plate, 150 g) |  |  |  |  |  |  |  |  |  |
| 175 | Green beans (1 plate, 200g) |  |  |  |  |  |  |  |  |  |
| 176 | Parsnips (65g), turnips (110g), swedes (60g) |  |  |  |  |  |  |  |  |  |
| 177 | Onions (1/2 unit, 50g) |  |  |  |  |  |  |  |  |  |
| 178 | Garlic (1 clove, 10g) |  |  |  |  |  |  |  |  |  |
| 179 | Mushrooms, chanterelles (1 small cooked plate, 100g) |  |  |  |  |  |  |  |  |  |
| 180 | Sweet peppers (1 medium size, 160g) |  |  |  |  |  |  |  |  |  |
| 181 | Beansprouts (60g) |  |  |  |  |  |  |  |  |  |
| 182 | Endives, lettuce, celery (1 plate, 150 g) |  |  |  |  |  |  |  |  |  |
| 183 | Cucumber (1 plate, 150 g) |  |  |  |  |  |  |  |  |  |
| 184 | Watercress (20g) |  |  |  |  |  |  |  |  |  |
| 185 | Tomatoes (1 unit, 85g) |  |  |  |  |  |  |  |  |  |
| 186 | Sweetcorn, kernels (60g) |  |  |  |  |  |  |  |  |  |
| 187 | Beetroot (1/2 unit, 75g) |  |  |  |  |  |  |  |  |  |
| 188 | Boiled, roasted potatoes (1 serving, 150g) |  |  |  |  |  |  |  |  |  |
| 189 | French fries (1 serving, 150g) |  |  |  |  |  |  |  |  |  |
| 190 | Sweet potato, cassava (1 serving, 150g) |  |  |  |  |  |  |  |  |  |
| 191 | Algae (nori, seaweed etc.) (1-3g dry equal to heaped teaspoon of Nori flakes or1.5 Nori leafs) |  |  |  |  |  |  |  |  |  |
|  | **LEGUMES** (in this group, you can include pasta or flour made from **100%** lentils, chickpeas or other legumes in the corresponding item)  (60g in raw or 1 plate boiled, 150 g) |  |  |  |  |  |  |  |  |  |
| 192 | Dried beans e.g., navy, lima, kidney, black, pinto, black eye |  |  |  |  |  |  |  |  |  |
| 193 | Chickpeas |  |  |  |  |  |  |  |  |  |
| 194 | Dried soybean e.g., mature, mung |  |  |  |  |  |  |  |  |  |
| 195 | Lentils e.g., brown, green, yellow, red lentils |  |  |  |  |  |  |  |  |  |
| 196 | Split peas |  |  |  |  |  |  |  |  |  |
| 197 | Shelled edamame pods (green soybeans) (80g in raw) |  |  |  |  |  |  |  |  |  |
| 198 | Other vegetable (1 plate, 200g); legume (60g in raw or 1 plate boiled, 150 g) |  |  |  |  |  |  |  |  |  |
|  | 1. |  |  |  |  |  |  |  |  |  |
|  | 2. |  |  |  |  |  |  |  |  |  |

|  |  | **AVERAGE USE LAST YEAR** | | | | | | | |  |
| --- | --- | --- | --- | --- | --- | --- | --- | --- | --- | --- |
|  | **FOODS AND AMOUNTS** | **Never or less than once a month** | **1-3 per month** | **Once a week** | **2-4 per week** | **5-6 per week** | **Once a day** | **2-3 per day** | **4-5 per day** | **6+ per day** |
|  | **FAT AND OILS**  as a spread, bread dipping, for seasoning, or in salads |  |  |  |  |  |  |  |  |  |
| 199 | Butter  (1 teaspoon, 5g) |  |  |  |  |  |  |  |  |  |
| 200 | Margarine (1 teaspoon, 5g) |  |  |  |  |  |  |  |  |  |
|  | Vegan margarine (100% plant-based) (1 teaspoon, 5g) |  |  |  |  |  |  |  |  |  |
| 201 | Lard/dripping (1 tablespoon, 10g) |  |  |  |  |  |  |  |  |  |
|  | **Vegetable oils** |  |  |  |  |  |  |  |  |  |
| 202 | Olive oil (1 tablespoon) |  |  |  |  |  |  |  |  |  |
| 203 | Sunflower oil (1 tablespoon) |  |  |  |  |  |  |  |  |  |
| 204 | Soybean oil (1 tablespoon) |  |  |  |  |  |  |  |  |  |
| 205 | Rapeseed oil (1 tablespoon) |  |  |  |  |  |  |  |  |  |
| 206 | Peanut oil (1 tablespoon) |  |  |  |  |  |  |  |  |  |
| 207 | Flaxseed oil (1 tablespoon) |  |  |  |  |  |  |  |  |  |
| 208 | Coconut oil (1 tablespoon) |  |  |  |  |  |  |  |  |  |

|  |  | **AVERAGE USE LAST YEAR** | | | | | | | |  |
| --- | --- | --- | --- | --- | --- | --- | --- | --- | --- | --- |
|  | **FOODS AND AMOUNTS** | **Never or less than once a month** | **1-3 per month** | **Once a week** | **2-4 per week** | **5-6 per week** | **Once a day** | **2-3 per day** | **4-5 per day** | **6+ per day** |
| 209 | FOOD THAT WAS FRIED AT HOME |  |  |  |  |  |  |  |  |  |
| 210 | FRIED FOOD AWAY FROM HOME |  |  |  |  |  |  |  |  |  |
| 211 | GRILLED OR ROAST MEAT |  |  |  |  |  |  |  |  |  |

Did you regularly use fortified salt **during the past year?**

1. Yes
2. No
3. Sometimes
4. Don't know

If the answer is **Yes or Sometimes**, please select **ONLY ONE** option you **usually use**; then tick (✓) **ONE option** in frequency.

|  |  | **AVERAGE USE LAST YEAR** | | | | | | | |  |
| --- | --- | --- | --- | --- | --- | --- | --- | --- | --- | --- |
|  |  | **Never or less than once a month** | **1-3 per month** | **Once a week** | **2-4 per week** | **5-6 per week** | **Once a day** | **2-3 per day** | **4-5 per day** | **6+ per day** |
|  | 1. Fortified with iodine 2. Fortified with fluoride 3. Fortified with folic acid 4. Fortified with fluoride and folic acid 5. Other (specify): |  |  |  |  |  |  |  |  |  |

**Have you taken any vitamins, minerals, multivitamin/multimineral supplements, DHA/EPA/omega-3/fish-oil supplements, amino acids, other food supplements during the past year?**

1. Yes
2. No
3. Sometimes
4. Don't know

If answer is Yes or Sometimes, please complete the following table on supplements, fill in the brand, dose and amount for those you usually use; then tick (✓) **ONE** option in **frequency** for how often you use the supplements. You have an example.

| All nutrients | Brand | Dosage (dosage of the supplement for each tablet, drop, capsule or teaspoon) | Amount (number of tablets, capsules, drops or teaspoons that you consume) | Frequency |
| --- | --- | --- | --- | --- |
| ***Example:***  *Vitamin D3*  *Cholecalciferol* | *SOLGAR* | *4000 IU (100 µg)* | *1 capsule* | *Once a day* |
|  |  |  |  | 1. Never or less than once a month 2. 1-3 per month 3. Once a week 4. 2-4 per week 5. 5-6 per week 6. Once a day |
|  |  |  |  | 1. Never or less than once a month 2. 1-3 per month 3. Once a week 4. 2-4 per week 5. 5-6 per week 6. Once a day |
|  |  |  |  | 1. Never or less than once a month 2. 1-3 per month 3. Once a week 4. 2-4 per week 5. 5-6 per week 6. Once a day |
|  |  |  |  | 1. Never or less than once a month 2. 1-3 per month 3. Once a week 4. 2-4 per week 5. 5-6 per week 6. Once a day |
|  |  |  |  | 1. Never or less than once a month 2. 1-3 per month 3. Once a week 4. 2-4 per week 5. 5-6 per week 6. Once a day |
|  |  |  |  | 1. Never or less than once a month 2. 1-3 per month 3. Once a week 4. 2-4 per week 5. 5-6 per week 6. Once a day |
|  |  |  |  | 1. Never or less than once a month 2. 1-3 per month 3. Once a week 4. 2-4 per week 5. 5-6 per week 6. Once a day |
|  |  |  |  | 1. Never or less than once a month 2. 1-3 per month 3. Once a week 4. 2-4 per week 5. 5-6 per week 6. Once a day |
| Others such as Food/Herbal supplements (specify below): |  |  |  |  |
| 1. |  |  |  | 1. Never or less than once a month 2. 1-3 per month 3. Once a week 4. 2-4 per week 5. 5-6 per week 6. Once a day |
| 2. |  |  |  | 1. Never or less than once a month 2. 1-3 per month 3. Once a week 4. 2-4 per week 5. 5-6 per week 6. Once a day |
| 3. |  |  |  | 1. Never or less than once a month 2. 1-3 per month 3. Once a week 4. 2-4 per week 5. 5-6 per week 6. Once a day |
